# Well-defined alginate oligosaccharides ameliorate joint pain and inflammation in a mouse model of gouty arthritis

**DOI:** 10.7150/thno.95611

**Published:** 2024-05-19

**Authors:** Chengyu Yin, Qianqian Lyu, Zishan Dong, Boyu Liu, Keke Zhang, Zhende Liu, Qing Yu, Peiyi Li, Zhuoqun Wei, Yan Tai, Chuan Wang, Jianqiao Fang, Weizhi Liu, Boyi Liu

**Affiliations:** 1 Department of Neurobiology and Acupuncture Research, The Third Clinical Medical College, Key Laboratory of Acupuncture and Neurology of Zhejiang Province, Zhejiang Chinese Medical University, Hangzhou, China.; 2 Fang Zongxi Center, MoE Key Laboratory of Marine Genetics and Breeding, College of Marine Life Sciences, Ocean University of China, Qingdao, China.; 3 Laboratory for Marine Biology and Biotechnology, Qingdao Marine Science and Technology Center, Qingdao, China.; 4 Department of Pharmacology, Hebei Medical University, Shijiazhuang, China.; 5 Haitang (Jiangsu) Biotechnology Co, Ltd., Nantong, Jiangsu, China.; 6 Academy of Chinese Medical Sciences, Zhejiang Chinese Medical University, Hangzhou, China.

**Keywords:** NLRP3, Inflammation, ROS, TRPV1, Oligosaccharides, Neuropeptide

## Abstract

**Background:** Gouty arthritis causes severe pain and inflammation. Alginate oligosaccharides (AOSs) are natural products derived from alginate and have anti-inflammatory properties. We explored the potential effects of AOSs with different degrees of polymerization (Dp) on gouty arthritis and associated mechanisms.

**Methods:** We established a mouse model of gouty arthritis by injecting monosodium urate (MSU) into ankle joint. Nocifensive behavior, gait and ankle swelling were used to study AOS's effects. Biochemical assays, *in vivo* imaging, live cell Ca^2+^ imaging, electrophysiology, RNA-sequencing, etc. were used for mechanism exploration.

**Results:** AOS2 (Dp=2), AOS3 (Dp=3) and AOS4 (Dp=4) all inhibited ankle swelling, whereas AOS2&3 produced the most obvious analgesia on model mice. AOS3, which was picked for further evaluation, produced dose-dependent ameliorative effects on model mice. AOS3 reversed gait impairments but did not alter locomotor activity. AOS3 inhibited NLRP3 inflammasome activation and inflammatory cytokine up-regulation in ankle joint. AOS3 ameliorated MSU-induced oxidative stress and reactive oxygen species (ROS) production both *in vivo* and *in vitro* and reversed the impaired mitochondrial bioenergetics. AOS3 activated the Nrf2 pathway and promoted Nrf2 disassociation from Keap1-bound complex and Nrf2 nuclear translocation, thus facilitating antioxidant gene expression via Nrf2-dependent mechanism. *Nrf2* gene deficiency abolished AOS3's ameliorative effects on pain, inflammation and oxidative stress in ankle joints of model mice. AOS3 reduced TRPV1 functional enhancement in DRG neurons and constrained neuroactive peptide release.

**Conclusions:** AOS3 ameliorates gouty arthritis via activating Nrf2-dependent antioxidant signaling, resulting in suppression of ROS-mediated NLRP3 inflammasome activation and TRPV1 enhancement. AOS3 may be novel therapeutics for gouty arthritis.

## Introduction

Gouty arthritis is initiated by deposition of monosodium urate in the joints, which causes an intense inflammatory response in the joint and periarticular tissues 1, 2. It represents one of the most common types of inflammatory arthritis worldwide 1, 3. It brings in severe pain and inflammation in the joints that dramatically affects the life quality of patients 4. With the aging population and dietary changes, incidence of gouty arthritis has been increasing 5. Colchicine and non-steroid anti-inflammatory drugs (NSAIDs) are first-line medications for relieving gout flares 6. However, constantly taking these medicines can result in serious adverse effects, especially among people with comorbidities like chronic kidney disease, gastrointestinal ulcers and diabetes 4, 7, 8. Therefore, exploring novel therapeutic options for gouty arthritis is of important clinical significance.

When excessive MSU deposits to the joint, resident macrophages are the first immune cells that respond 9. The engulfment of MSU crystals by macrophages triggers the overproduction of pro-inflammatory cytokines that further recruit neutrophil infiltration 9, 10. The pathophysiology of gouty arthritis largely depends on NLRP3 inflammasome signaling, the activation of which triggers caspase-1-mediated IL-1β maturation and extracellular release 11, 12. Meanwhile, during gouty arthritis, large amounts of reactive oxygen species (ROS) are produced in the inflamed joint tissues 13-16. ROS make important contributions to the activation process of NLRP3 inflammasome 17, 18. At present, NLRP3 inflammasome and IL-1β production are considered the key machineries leading to joint inflammation that contributes to pain and inflammation of gouty arthritis 19.

**Figure 1:**
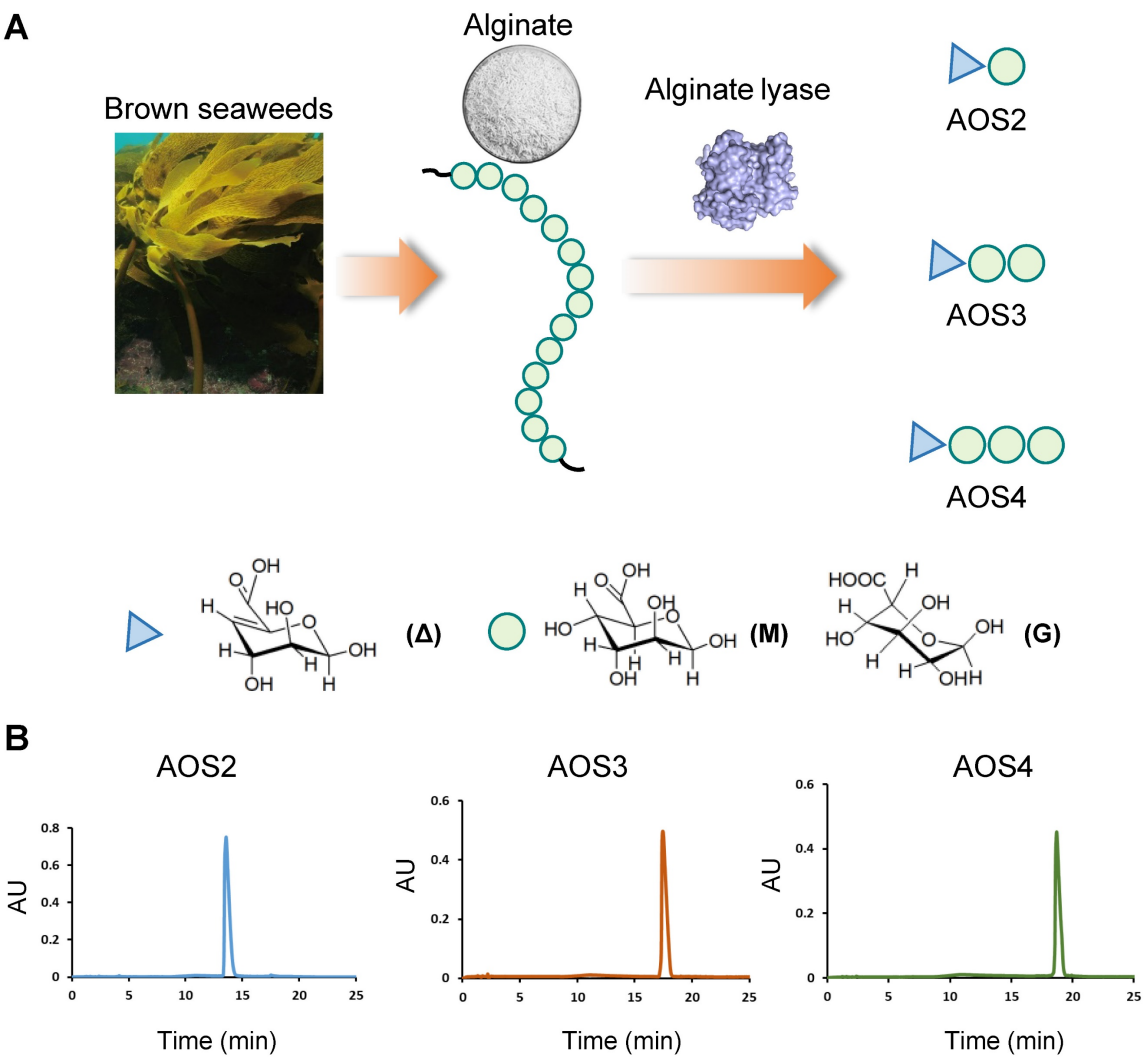
** Characterization of AOSs used in this study.** (A) Schematic picture showing that AOSs used in this study were prepared by alginate lyases degradation of alginates. Alginate lyases depolymerize alginate through a β-elimination reaction leading to the formation of unsaturated residues, 4-deoxy-L-erythro-hex-4-enopyranosyluronic residues (symbolized by Δ), at the nonreducing end of the products. Circle indicated the M or G unit in alginate. (B) HPLC analysis showing that AOS2-4 (AOS with Dp2-4) were homogenized with specific Dp.

Alginates are linear polysaccharides extracted from brown seaweeds, accounting for one-third of the polysaccharides in the ocean. They consist of α-L-guluronic acid (G) and its C-5 epimer β-D-mannuronic acid (M), with distinct mixed ratio and degree of polymerizations (Dps) 20 (Figure 1A). Alginate oligosaccharides (AOSs) are natural products derived from chemical or enzymatic degradation of alginates. The degradation of alginate into AOSs further enhances solubility and reduces molecular weight, which promotes explorations of AOSs' potential benefits for health 21. Recent studies unraveled that AOSs possess multiple biological activities, including anti-neurodegeneration, anti-tumor, anti-inflammation as well as immune regulations, etc. Some of these biological effects have been proven to be influenced by the Dp of AOSs. 21, 22. However, AOSs with multiple Dps were used in the majority of current biological activity investigation due to the lack of homogenized AOSs 22, 23. Up-to-date, no investigations have ever explored the potential effects of AOSs on pain, especially joint pain elicited by gouty arthritis. Therefore, in this study, the homogenized AOSs with specific Dp were used to determine their potential therapeutic effects on a mouse gouty arthritis model. The related mechanisms were further investigated.

## Methods

### Animals

C57BL/6J mice (male, 6-8 weeks old) were purchased from Shanghai Laboratory Animal Center of Chinese Academy of Sciences. Ly6G-IRES-GFP knock-in mouse line and *Nrf2* knockout mouse line were generated via Cas9-associated guide RNA (gRNA) technique by Shanghai Model Organisms Center, Inc. (Shanghai, China). Animals were housed in the Laboratory Animal Center of Zhejiang Chinese Medical University. Food and water were available *ad libitum*. Five mice were housed per cage. All experiments were conducted with the approval of the Laboratory Animal Management and Welfare Ethical Review Committee of Zhejiang Chinese Medical University (Protocol No.: IACUC-20201214-13).

### Gouty arthritis model establishment

MSU (Merck, USA) suspension was prepared in endotoxin-free PBS and then intra-articular injected at a dosage of 0.5 mg/20 μl into the right ankle joint of the mice to establish gouty arthritis model as previously described 24. The control mice received an equal volume of PBS injection. The mice were anesthetized with 2.5% isoflurane during the injection. Ankle swelling and mechanical hypersensitivity, which would occur 2 h after MSU injection, were regarded as criteria for successful establishment of the model as reported 14.

### Reagents and administration

The AOSs used in this study were provided by Haitang (Jiangsu) Biotechnology Co, Ltd. Briefly, alginate lyases were utilized to depolymerize alginate resulting a mixture of AOSs. After purification, three kinds of AOSs with specific Dp were prepared, i.e. AOS2 (Dp=2), AOS3 (Dp=3) and AOS4 (Dp=4). HPLC analysis indicated that the homogeneity of AOS2-4 towards their Dps exceeded 95% (Figure 1B). The molecular masses of AOS2-4 were further determined by mass spectrometry (MS) and shown in Figure S1. To investigate ankle swelling and analgesic effect, AOS2, AOS3 and AOS4 were administered at a standardized dosage of 200 mg/kg, along with varying dosage levels of AOS3 (100, 200 or 400 mg/kg), indomethacin (10 mg/kg), or a corresponding vehicle (PBS). These substances were administered intraperitoneally (i.p) 1 h prior to MSU injection and again at 5 and 23 h post-injection, for a total of 3 administrations. In cases where 48 h time point was evaluated, AOS3 were further administered at 47 h time points. To assess the effect of AOSs after model establishment via oral rout, AOS3 (200 mg/kg) was administered via oral gavage (o.g.) at 5 and 23 h time points after MSU injection.

### Mechanical allodynia assessment

Mechanical allodynia was determined by von Frey tests as previously described 25. In brief, mice were individually habituated in an opaque Plexiglas chamber on a wire mesh platform for 30 min prior to test. The testing involved the application of a series of von Frey filaments (0.008-2 g, UGO Basile, Italy) perpendicularly to the heel region of the plantar surface of the hind paw for a maximum of 5 seconds, from underneath. A positive response was recorded when there was a sudden withdrawal of the paw, along with licking or vigorous shaking in response to the stimulus. The 50% PWT was determined by the “Up-Down” testing paradigm through Dixon test 26, 27.

### Heat hyperalgesia assessment

Heat hyperalgesia was evaluated by Hargreaves test. A radiant light beam generated by a light bulb was directed to the right hind paw of mice to measure paw withdrawal latency (PWL) via the Plantar Test Apparatus (Ugo Basile, Italy). A cutoff threshold of 20 s was set to avoid tissue injury. The heat stimulation was repeated 3 times with 5 min interval for each trial, and the mean PWL was calculated.

### Determination of the ankle swelling

Ankle edema was measured with a digital caliper with 0.1 mm accuracy before and after ankle joint injection of MSU crystals. The degree of swelling were presented as a percentage increase in ankle joint diameter, and calculated as follows: % increase in ankle diameter = (D_after_-D_basal_)/D_basal_. Each mouse was measured 3 times, and the mean value was calculated.

### Gait analysis

The gait of mice was monitored through the DigiGait imaging and analysis system (MouseSpecifics, Inc., USA) as we recently described 28. The mouse was placed in the open end of an enclosed glass platform illuminated with red ceiling light-emitting diodes and allowed to walk voluntarily through the walkway. As the mice walked across the glass floor, a high-speed camera positioned beneath the apparatus captured images of each paw's illuminated area and transmitted the data to the gait analysis software. Valid data consisted of a minimum of 3 sequential step cycles or complete passes through the tunnel. For this study, 3 parameters were utilized to evaluate gait impairments: (1) “paw area” representing the area of hind paw contact with the glass plate; (2) “stride length” indicating the duration of ground contact for a single hind paw without the contralateral hind paw touching the glass plate; (3) “swing” denoting the duration of hind paw non-contact with the glass plate.

### Rotarod test

Locomotor function was assessed using the Rotarod system (Panlab, Spain) as previously described 29. The mice underwent 3 separate trials with a 10-min interval between each trial. The speed of the rotation gradually increased from 5 to 40 rpm over a 5-minute duration. The latency of falling was recorded thereafter.

### Open field test

Mice were placed in the center of an open field arena with a size of 45 cm (length) × 45 cm (width) × 30 cm (height). The mice were all put in the testing room for 30 min before the test to acclimate to the testing environment. The distance the animals traveled within a 10-min timeframe was recorded using ANY-maze software (Stoelting, IL, USA) and analyzed thereafter. All behavioral tests were conducted in a blinded manner during daytime. In all experiments, the experimenters were unaware of the treatments or experimental manipulations.

### Myeloperoxidase (MPO) activity assay

Neutrophil recruitment in the ankle joint of mice from each group was evaluated by quantifying of the activity of the enzyme MPO, using a commercially available MPO Assay Kit (Elabscience, China) according to the manufacturer's instructions. The ankle joint was homogenized and centrifuged at 10,000 × g at 4 °C for 15 min. 10 μl of the supernatant was transferred into PBS (pH 6.0) containing 0.17 mg/ml 3,3',5,5'-tetramethylbenzidine and 0.0005% H_2_O_2_. MPO catalyzed the redox reaction of H_2_O_2_ and 3,3',5,5'-tetramethylbenzidine and produced yellow colored compounds, and the absorbance at 460nm was determined using a microplate reader (SpectraMax M4, Molecular Devices, CA, USA). MPO activity was calculated and expressed as U/g tissue.

### RNA Sequencing (RNA-Seq) and bioinformatics analysis

Ankle joint tissues were collected from mice in each group 24 h after MSU injection. RNA isolation was performed using Trizol reagent (Invitrogen, Carlsbad, USA) following the manufacturer's instructions. Quantification and integrity analysis of the total RNA were conducted using a NanoDrop Spectrophotometer (Thermo, USA) by running 1 µl of each sample. RNA-Seq libraries were constructed and sequenced by BGISEQ-500 (BGI, China). After quality control, the raw RNA-Seq data were filtered to obtain the clean data used for alignment to the mouse genome (Mus musculus GRCm38.p5, NCBI). Based on these read counts, the "DESeq" R package (1.10.1) was utilized for differential expression analysis between the two groups. Differentially expressed genes (DEGs) were determined as those with an adjusted P value (q value) less than 0.05 and fold change larger than 1.5 (up-regulation) or smaller than 2/3 (down-regulation). The cluster analysis and heatmap visualization of gene expression patterns were performed using the TBtools software 30. Gene Ontology (GO) enrichment analysis in the molecular function category and KEGG pathway enrichment analysis were visualized by "ClusterProfiler" package in Rstudio software. In order to identify the connectivity degree of each proteins, the web-based STRING database (http://string-db.org/) was used to produce PPI predictions after uploading the union gene list to the search bar. Based on the interplay relationships, a PPI network was constructed and visualized using Cytoscape software. CytoHubba plugin was utilized to identify hub genes with significant connectivity within the network. CytoHubba was also used to calculate the interaction scores of each protein node based on its interactions with other nodes in the network. Single-sample gene set enrichment analysis (ssGSEA) was performed using the "GSVA" package in R software on a metagene set of 28 immune cells as previously described 31. The calculated "Expression" value served as a quantitative measure to demonstrate the enrichment level of metagenes in each sample, reflecting the intensity of infiltration of innate immune cell types corresponding to the metagenes.

### Histopathological assessment of ankle joint

Ankle joints were fixed with 4% paraformaldehyde in PBS, and then decalcified for 3 weeks with EDTA. The specimens were subsequently embedded in paraffin for histological analysis. Hematoxylin and eosin (H&E) staining was used for analysis, and morphological evaluation was performed under a light microscope with 10× and 40× objectives. The number of infiltrated inflammatory cells per observation field in each group were counted in a blind manner.

### Co-immunoprecipitation (co-IP)

Raw264.7 macrophage cells were lysed in RIPA buffer (Beyotime, China) containing a protease inhibitor mixture (Roche, USA) after 4 h treatment with AOSs. The nuclear fractions of cells were extracted using a nuclear protein extraction kit (Absin, China) according to manufacturer's instructions. The procedures were performed using Immunoprecipitation Kit with Protein A+G Magnetic Beads (Beyotime, USA) following the manufacturer's instructions. Briefly, lysates of nuclear fractions were centrifuged at 13,000 g for 10 min at 4 °C, and 5% of the supernatant was taken for a whole-cell lysate sample. Immunoprecipitation was carried out by adding anti-Keap1 antibody to protein lysates. The samples were gently rocked overnight, and subsequently incubated with beads for 2 h at 4 °C. After being washed 3 times, the samples were subjected to Low-pH elution, boiled in the SDS sample buffer, and then analyzed using SDS-PAGE and immunoblotting.

### Western blot

Western blot analysis was performed following previously reported methods 32. Briefly, ankle joint tissues were isolated, and snap-frozen in liquid nitrogen and stored at -80 °C. The tissues were then homogenized in RIPA lysis buffer with protease inhibitor using Bullet Blender (BBX24, NextAdvance Inc., NY, USA) at 4 °C for 20 min at maximum speed. After centrifugation at 15, 000 g for 12 min at 4 °C, the resulting supernatants were collected and protein concentration was quantified using a BCA Protein Assay Kit (Thermo, MA, USA). A total of 30 μg protein was loaded in each lane of 8% or 15% SDS-PAGE gels. Subsequently, the proteins were transferred onto polyvinyl difluoride (PVDF) membranes (Millipore, Germany). The membranes were blocked with 5% non-fat milk at room temperature for 2 h, followed by overnight incubation at 4 °C with the following primary antibodies diluted in blocking buffer: IL-1β (1:1,000, rabbit polyclonal, #ab9722, Abcam, UK), NLRP3 (1:1,000, rabbit polyclonal, #NBP2-12446, Novus, USA), caspase-1 (1:500, rabbit polyclonal, #ab1872, Abcam, UK), Nrf2 (1:1,000, rabbit polyclonal, #R1312-8, HuaBio, China), Keap1 (1:1,000, rabbit polyclonal, #4678S, CST Signaling, USA), ASC (1:1,000, rabbit polyclonal, #ab309497, Abcam, UK) and β-actin (1:5,000, mouse monoclonal, #HA721186, HuaBio, China). Subsequently, these blots were incubated with HRP-conjugated second antibodies (1: 5000, CST Signaling, PA, USA) for 2 h at room temperature. The expression levels of targeted protein are normalized to the density of β-actin. The protein bands were detected using enhanced chemiluminescence regents (Biosharp, China) via FluoChem R System (Biotechne, USA). Quantitative analysis was performed with ImageJ (NIH, USA).

### Determination of oxidant/antioxidant status of ankle joint

Oxidative stress in the ankle joint was assessed by measuring the levels of various biochemical markers such as malondialdehyde (MDA), superoxide dismutase (SOD), glutathione (GSH), and H_2_O_2_. Samples were chopped and centrifuged and then the supernatant was collected for SOD, GSH, MDA, and H_2_O_2_ assays using commercially available kits, as previously described in our studies 12. SOD and GSH assaying kits were from Nanjing Jiancheng Bioengineering Institute (Nanjing, China). MDA and H_2_O_2_ assaying kits were from Beyotime (Shanghai, China).

### *In vivo* ROS imaging

The level of reactive oxygen species (ROS) in the ankle joints was measured using chemiluminescence 24 h after MSU injection. During bioluminescent imaging, mice were anesthetized with 2.5% isoflurane. Mice were intravenously injected with 25 mg/kg fluorescent ROS indicator L-012 sodium salt (Tocris, USA), and immediately imaged and recorded by IVIS Lumina LT *in vivo* imaging system (PerkinElmer, USA), as described previously 13. The luminescence signal intensity of each group mice was quantified using Living Image software (PerkinElmer, USA).

### Cell culture and determination of oxidant/antioxidant status

Raw264.7 macrophage cell line was purchased from ATCC (Shanghai, China) and maintained in DMEM (Gibco, USA) supplemented with 10% FBS (Hyclone, USA), 100 U/mL penicillin and 0.1 mg/mL streptomycin. Bone marrow-derived macrophages cells (BMDMs) were isolated from C57BL/6 mice. BMDMs were obtained from the tibia and femoral bones of mice as described before and plated at a density of 2 × 10^6^ cells in 6-well plates 33. The cells were cultured in 2 mL PRMI 1640 media containing 10% FBS and 30 ng/ml M-CSF for a period of 6 days. Medium was renewed every 3 days, after washing the cells 3 times with PBS, until complete differentiation of BMDMs was observed. Cells were maintained at 37 °C in a humidified atmosphere of 5% CO_2_ and 95% air.

Raw264.7 cells were seeded at 5×10^6^ cells/ml in 6-well plate and treated with 1 mg/ml AOSs for 30 min prior to the addition of MSU (500 μg/ml). The cells were collected after 4 h of stimulation with MSU and homogenized to measure the level of cellular oxidative stress. The SOD activity, GSH activity and MDA level were determined using commercially available kits as mentioned before. All procedure were conducted according to the manufacturer's instructions and analyzed with a microplate reader (Varioskan™ LUX, Thermo Scientific, USA) at specific excitation and emission wavelengths.

Intracellular ROS production was monitored by fluorescent probe 2′, 7′-dichloro-fluorescein diacetate (DCF-DA, 10 μM, Beyotime, China). The cells (5 × 10^6^) were seeded onto each well of 12-well plate and treated with 10 μM DCF-DA for 30 min at 37 °C. After incubation, the cells were washed 3 times with DMEM. Fluorescence and corresponding bright field pictures were acquired with a multifunctional fluorescence microscope (Axio Observer A1, Zeiss, Germany) with 488 nm excitation and 525 nm emission wavelengths. Uniformed microscope setting was maintained throughout all image capturing sessions and quantitative analysis of images was performed with Fiji (NIH, USA). For quantification of DCF staining with microplate reader, fluorescence intensities of each well in a 96-well plate (containing 1 × 10^5^ cells/well) were measured using a microplate reader (Varioskan™ LUX, Thermo Scientific, USA) with 485 nm excitation and 525 nm emission wavelengths. The fluorescence intensity was captured via SkanIt (Thermo Scientific, USA) and analyzed with Excel (Microsoft, USA).

### qPCR

Total RNA from the ankle joint tissues was extracted using TRIzol reagent (Invitrogen, USA). 1 μg of total RNA was reverse transcribed into cDNA using Prime ScriptTM RT reagent Kit (TaKaRa Bio Inc, China). Each process was carried out in triplicate with a 10 μl reaction mixture (containing 1 μl of cDNA and 10 μM of gene-specific primers) using a Fast Start Universal SYBR Green Master Kit (TaKaRa Bio Inc, China). Quantitative PCR was conducted on a LightCycler 480 real-time PCR system (Roche, USA). The mRNA expression levels were analyzed using the 2^-ΔΔCt^ method 34, 35 and were normalized to the mRNA level of *Actb*. The forward and reverse primers (Sangon Biotech, China) are listed in Table S1.

### Mitochondrial bioenergetics determination

Mitochondrial bioenergetics was measured in Raw264.7 macrophage cells using Seahorse XFe96 analyzer (Agilent, US) as we recently described 16. Cells were seeded at a density of 20,000 cells/well in Seahorse Bioscience XFe96 cell culture plates. After treatment with MSU (0.5 mg/ml) or vehicle (PBS) for 4 h and subsequent washes, cells were incubated in an unbuffered assay medium (DMEM, 5.5 mM glucose, 10 mM galactose, 2 mM L-glutamine, 1 mM sodium pyruvate, 100 U/mL penicillin, 100 μg/mL streptomycin, pH 7.4) at 37 °C in a non-humidified, CO_2_-free incubator. After establishing OCR in basal respiration (i.e., the respiration prior to the addition of any modulators), a Seahorse XF Cell Mito Stress Test profile was measured, which involved the determination of ATP-linked respiration by evaluating the OCR change upon addition of 1 μM oligomycin. Maximal respiration was achieved using 2.5 μM carbonyl cyanide-p-trifluoromethoxy-phenylhydrazone (FCCP), an uncoupler. Mitochondrial oxygen consumption was then halted by adding 1 μM rotenone and 0.5 μM antimycin A. The parameters related to mitochondrial bioenergetics were calculated based on the OCR readings.

### DRG neuron culture and Ca^2+^ imaging

L3-L5 DRGs were dissected in DMEM-F12 (Gibco, USA) and digested in collagenase/dispase solution at 37 °C for 1 h. DRG cells were triturated, centrifuged and re-suspended in DMEM-F12+10% FBS, and plated on sterile glass coverslips with Poly-D-Lysine coated. For Ca^2+^ imaging, cells were loaded with Fura-2AM (10 μM, Abcam, UK) for 45 min at 37 °C in the dark in the loading buffer (containing 140 NaCl, 5 KCl, 2 CaCl_2_, 2 MgCl_2_, and 10 HEPES, pH 7.4 with NaOH). After incubation, the cell culture was washed 3 times with loading buffer and prepared for ratiometric Ca^2+^ imaging. Ca^2+^ imaging was performed using the Nikon ECLIPSE Ti-S (Nikon, Japan) microscope. Polychrome V monochromator (Till Photonics, USA), equipped with Orca Flash 4.0 CCD camera (Hamamatsu, Japan), was used for excitation and image capture. MetaFluor (Molecular Devices, USA) was used for data processing. A cell was determined as a positively responding cell when the Ca^2+^ response reached > 20% of the baseline 36.

### Patch clamp recording

Whole-cell patch-clamp recordings were carried out in primary cultured DRG neurons with Axopatch-200B amplifier and Digidata 1550B digitizer (Axon Instruments, USA) as previously reported 37. Patch pipettes with a resistance of 3-5MΩ were fabricated using a pipette puller (P-97; Sutter Instruments, USA). The extracellular solution contained (mM): NaCl 150, KCl 5, CaCl_2_ 2.5, MgCl_2_ 1, glucose 10, and HEPES 10 (pH 7.4 with NaOH). The internal pipette solution contained (in mM): KCl 140, MgCl_2_ 1, CaCl_2_ 0.5, EGTA 5, HEPES 10, and ATP 3 (pH 7.4 with KOH). The action potentials (APs) were elicited by a 1000-ms inward current injection through the recording pipette under current clamp mode. Small diameter DRG neurons with Cm < 42 pF were recorded since a majority of these neurons are nociceptive 38, 39. Data were analyzed with Clampfit 10.2 (Axon Instruments).

### Statistical analysis

Results are expressed as mean ± SEM. Two-way analysis of variance (ANOVA) with repeated measures followed by Turkey's post-hoc test was performed to compare the effect of treatments at different time points. One-way followed by Tukey's post hoc test was used for comparison among groups ≥ 3. Statistical differences were considered to be significant at p < 0.05. Analyzes were performed by GraphPad Prism 9.

## Results

### AOSs attenuates ankle edema, mechanical allodynia and improves gait impairments in gouty arthritis model mice

**Figure 2:**
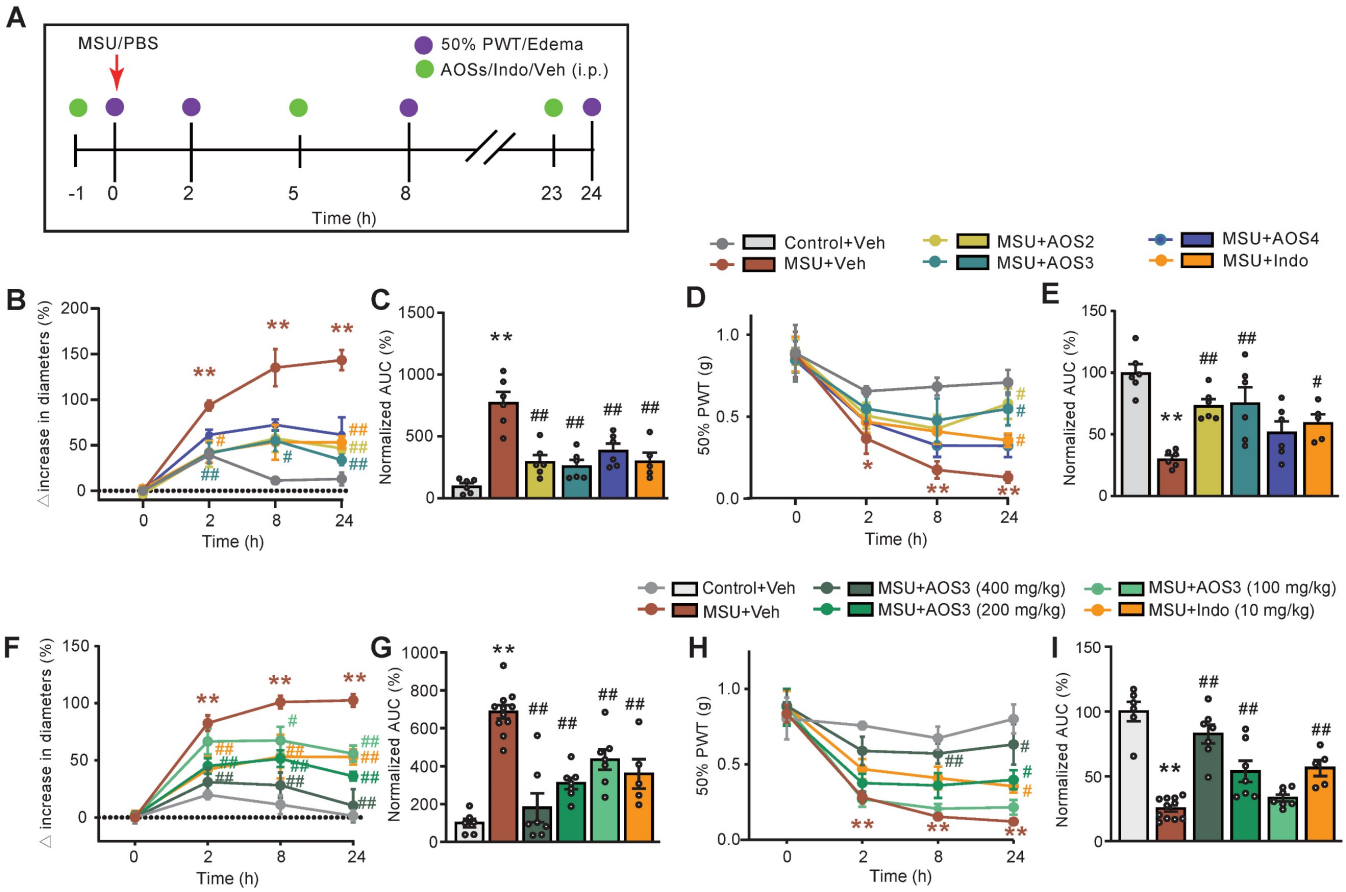
** Evaluation of the effects of AOSs on ankle edema and mechanical allodynia of a mouse model of gouty arthritis.** (A) Schematic protocol for experiments: PBS (20 μl) or MSU (0.5 mg/20 μl) was applied via intraarticular injection to ankle joint to establish control or gouty arthritis model, respectively. Different compositions (AOS2, AOS3 or AOS4, in 200 mg/kg dosage each), or different dosage (100, 200 or 400 mg/kg) of AOS3, indomethacin (Indo, 10 mg/kg) or corresponding vehicle was injected intraperitoneally (i.p.) at 1 h before and 5, and 23 h after model establishment. Mechanical allodynia and ankle edema were measured at time points as indicated. (B) Time courses of the effects of indomethacin (Indo) and different dosages of AOS3 on ankle edema. (C) Normalized AUC of panel B. (D) Time courses of the effects of indomethacin and different doses of AOS3 on mechanical allodynia of the hind paw. (E) Normalized AUC of panel D. (F) Time courses showing the effect of treatment with different compositions of AOS or indomethacin on ankle edema of MSU-treated mice. (G) Normalized AUC analysis of the curves shown in panel F. (H) Time courses showing the effect of treatment with different compositions of AOS or indomethacin on mechanical allodynia of MSU-treated mice. (I) Normalized AUC analysis of the curves shown in panel H. ^**^p<0.01, ^*^p<0.05 vs. Control+Veh group. ^#^p<0.05, ^##^p<0.01 vs. MSU + Veh group. n=5-11 mice/group. One-way ANOVA followed by Tukey's post hoc test was used in panel C, E, G&I. Two-way ANOVA followed by Tukey's post hoc test was used in panel B, D, F&H.

Intra-articular injection of MSU (500 μg/site) caused significant ankle swelling and mechanical allodynia, which commenced 2 h after the injection and continued for over 24 h compared to vehicle-treated control group (Figure 2A-E). These observations were consistent with previous literatures, indicating successful model establishment 12, 24. We then studied and compared the biological activities of AOSs with uniformed Dp. The effect of AOSs with Dp2, 3 or 4 (AOS2-4, respectively) on joint pain and inflammation of gouty arthritis model mice were examined. AOS was administered intraperitoneally (i.p.) 1 h before and 5, 23 h after MSU injection, for a total of 3 times (Figure 2A). Indomethacin (i.p.), a NSAID commonly used for gout flare alleviation, was used as positive control 6. Administration of AOS2, AOS3 or AOS4 (200 mg/kg) all significantly ameliorated joint inflammation (Figure 2B). Area under the curve (AUC) analysis of Figure 2B further depicted the anti-inflammatory effects of AOSs and indomethacin over the observation time course (Figure 2C). For mechanical allodynia, AOS2 and AOS3 produced the most effective alleviation, whereas AOS4 was not that effective (Figure 2D&E). We next chose AOS3 and further investigated its dose-response effect. We found that all 3 doses (100, 200, or 400 mg/kg, i.p.) of AOS 3 could reduce joint edema of model mice (Figure 2F&G). We then monitored AOS3's effect on mechanical allodynia of model mice. 200 or 400 mg/kg AOS3 produced obvious analgesic effect on model mice, whereas 100 mg/kg AOS3 was not effective (Figure 2H&I). As a control, indomethacin (10 mg/kg) significantly attenuated both pain and inflammation of ankle joint of model mice (Figure 2F-I). This finding indicates that AOSs can alleviate gouty arthritis pain and inflammation in model mice. We then chose AOS3 for subsequent studies.

We noticed in our recent studies that gouty arthritis model animals also developed obvious heat hyperalgesia 14, 40. AOS3 (200 mg/kg) treatment significantly alleviated heat hyperalgesia of model mice (Figure S2A&B). We further monitored the effects of AOS3 treatment on gouty arthritis developed at later time points (e.g. 48 h). AOS3 (200 mg/kg) treatment significantly reduced the mechanical allodynia and joint swelling 48 h after model establishment (Figure S2C-D). The effect of AOS3 on locomotor activity was evaluated since retardation in movement may result in unresponsiveness to mechanical stimuli. As shown in Figure S3A-C, AOS3 (200 mg/kg) treatment did not affect locomotor activity of mice tested by either open field or rotarod. We then evaluated if AOS3 was still effective when administered after model establishment. In the meantime, we also tested whether orally applied AOSs still exerted therapeutic effects. AOS3 (200 mg/kg) or vehicle (PBS) was delivered through oral gavage (o.g.) to mice at 5 and 23 h after establishing the model (Figure S4A). Oral administration of AOS3 significantly ameliorated mechanical allodynia in model mice after model was established (Figure S4B&C).

**Figure 3:**
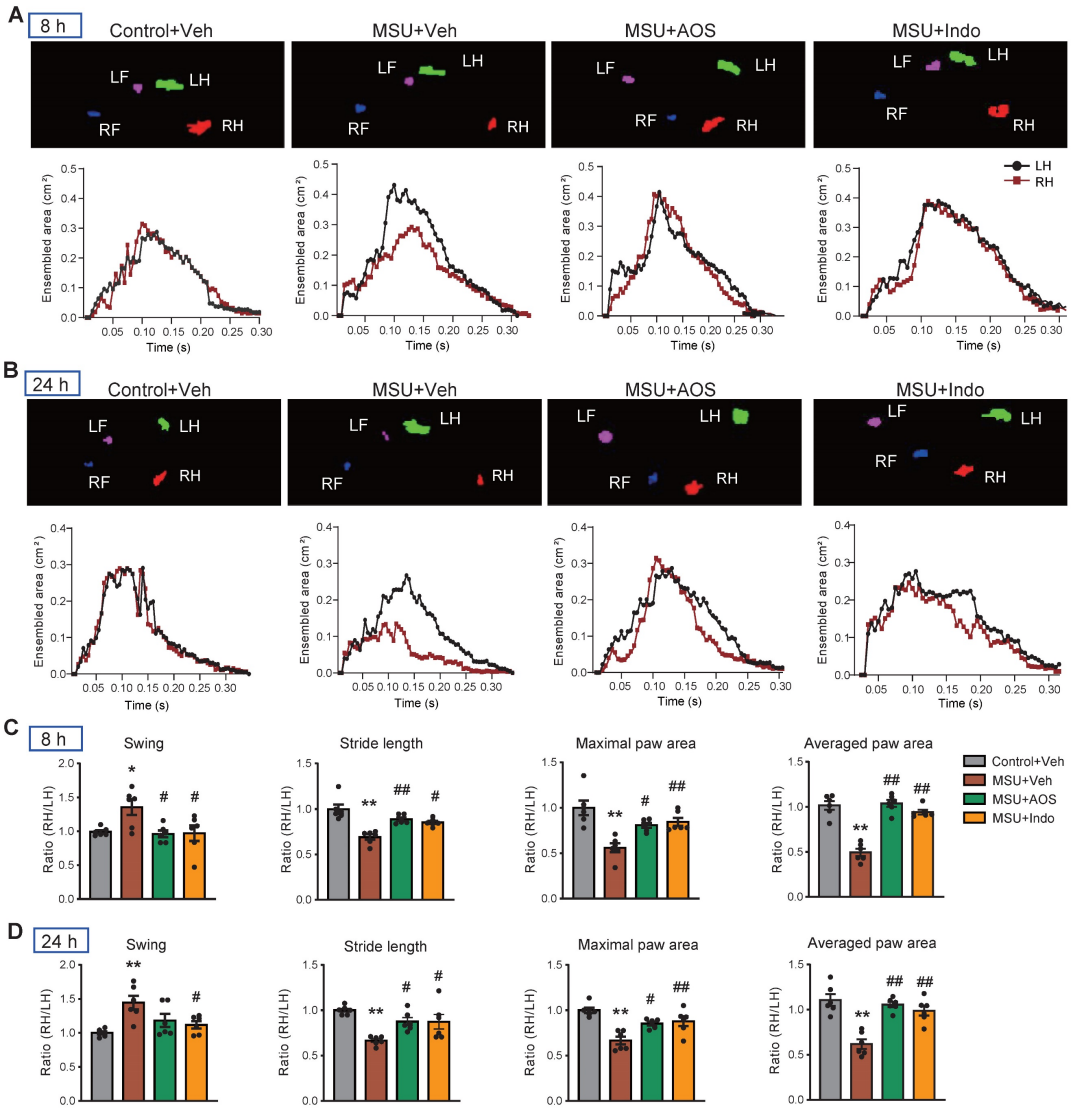
** AOS3 treatment improved gait impairments that were exhibited by gouty arthritis model mice.** (A&B) Representative pictures showing the mice under gait analysis at 8 (A) and 24 h (B) after model establishment. Top panel shows instant recording of paw images of the mice, whereas the bottom panel shows representative data for ensembled paw area of the left hind (LH) vs. right hind (RH) of mice from Control+Veh, MSU+Veh, MSU+AOS and MSU+Indomethacin groups at 8 h (A) or 24 h (B) time points. (C&D) Summary of swing ratio (RH/LH), stride length ratio (RH/LH) and paw area ratio (RH/LH) of 4 groups of mice at 8 (C) and 24 h (D) time points. n=6-7 mice group. ^*^p<0.05, ^**^p<0.01 vs. Control+Veh. ^#^p<0.05, ^##^p<0.01 vs. MSU+Veh group. One-way ANOVA followed by Tukey's post hoc test was used in panel C&D.

Gouty arthritis can lead to gait impairment that severely restricts mobility of patients' lower limbs and affects daily activities 41. Our recent studies demonstrate that MSU-induced gouty arthritis model mice display similar gait impairments 14, 16, 37. Therefore, we investigated whether AOS has the potential to correct gait impairments of model mice. Gait analysis revealed obvious gait impairments in model mice following MSU injection compared to control group. The maximal and averaged paw area ratios, swing ratio and stride length ratio were all significantly affected in model mice at 8 and 24 h after model establishment (Figure 3A-D). Notably, treatment with AOS3 (200 mg/kg) effectively reversed the impaired gait parameters at both 8 h and 24 h time points, yielding similar results to those achieved with indomethacin (Figure 3A-D). These findings indicate that AOS3 has the capability of improving gait impairments in gouty arthritis model mice.

### The identification of NLRP3 inflammasome signaling as the molecular target of AOS3 against gouty arthritis

**Figure 4:**
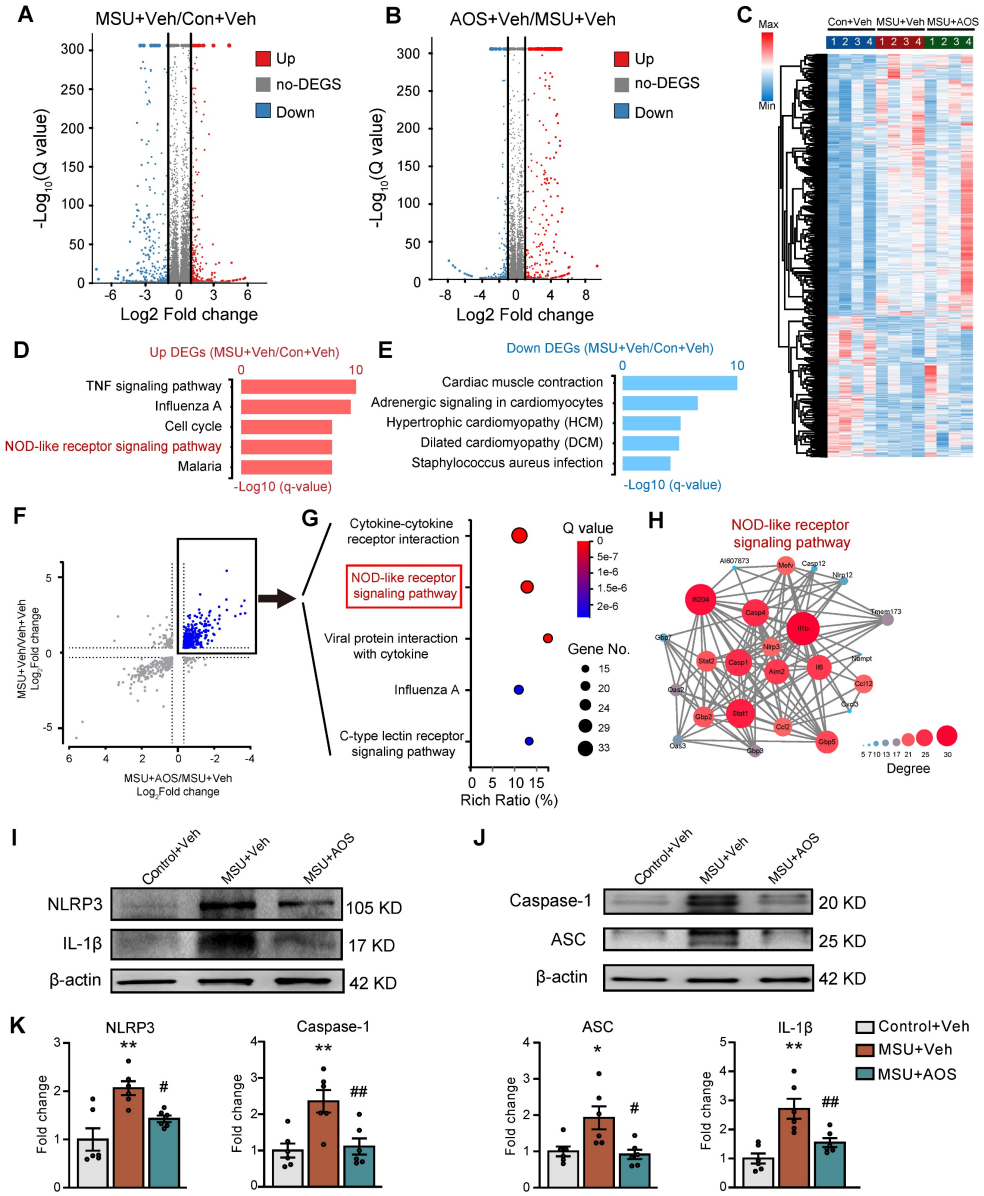
** Exploration of potential molecular targets of AOS3 in ankle joint tissues of gouty arthritis model mice by means of RNA-Seq and the validations.** (A&B) Volcano plots showing DEGs derived from comparisons between MSU+Veh vs. Control+Veh or AOS+Veh vs. MSU+Veh groups of mice. Red and blue dots show up- and down-regulated DEGs, respectively. Gray dots indicate non-DEGs. (C) Heat map with hierarchical clustered analysis showing the DEGs identified from panel A&B. (D&E) Bar charts showing the top 5 enriched pathways identified by Gene Ontology (GO) analysis of up- or down-regulated DEGs derived from MSU+Veh vs. Control+Veh group. (F) Scattered plot showing the DEGs that were overlapped between MSU+Veh/ Control+Veh group and AOS+Veh vs. MSU+Veh group. (G) Bubble plots showing the top 5 significant pathways of the DEGs (colored in blue in panel F and outlined by black box) identified by KEGG. (H) Construction of PPI network of DEGs allocated to NOD-like receptor signaling pathway as in panel G (outlined by the red box). Deeper color and larger circle indicate more protein-protein interactions and vice versa. n=4 mice/group for RNA-Seq. (I&J) Representative immunoblots of NLRP3, ASC, cleaved IL1β, cleaved Caspase1 in Control+Veh, MSU+Veh and MSU+AOS groups of mice. β-actin was used as loading control. (K) Column charts showing the quantifications of NLRP3, cleaved IL-1β, ASC and cleaved Caspase1 protein levels in ankle joint tissues of three groups. n=6 mice/group. ^*^p<0.05, ^**^p<0.01 vs. Control+Veh group. ^#^p<0.05, ^##^p<0.01 vs. MSU+Veh group. One-way ANOVA followed by Tukey's post hoc test was used in panel K.

To further analyze the mechanisms underlying therapeutic effects of AOS3, a high-throughput RNA-Sequencing (RNA-Seq) was performed for expression profiling. Compared with control group, gouty arthritis model mice receiving vehicle treatment (MSU+Veh group) exhibited 1,830 differentially expressed genes (DEGs), with 1,048 up-regulated and 782 down-regulated, in the inflamed ankle joint (Figure 4A). Compared with MSU+Veh group, gouty arthritis model mice receiving AOS3 treatment (MSU+AOS) showed 903 DEGs, with 484 up-regulated and 419 down-regulated, in the ankle joint (Figure 4B). These DEGs were illustrated by a heat map as shown in Figure 4C. Gene ontology (GO) analysis of up-regulated DEGs from MSU+Veh vs. Control+Veh revealed that the most significantly enriched biological processes include TNF signaling pathway, NOD-like receptor signaling pathway, cardiac muscle contraction, etc. (Figure 4D). The enriched pathways of down-regulated DEGs were shown in Figure 4E. We next screened the overall overlapping DEGs between MSU+AOS/MSU+Veh and MSU+Veh/Control+Veh groups (Figure 4F). We were especially interested in DEGs that were up-regulated in MSU+Veh/Control+Veh group but down-regulated in MSU+AOS/MSU+Veh group as indicated by black box in Figure 4F. KEGG analysis of this specific group of DEGs revealed that AOS3 treatment downregulated genes involved in cytokine-cytokine receptor interaction, NOD-like receptor signaling pathway, etc. in the inflamed ankle joint of gout model mice (Figure 4G).

Since the NOD-like receptor signaling pathway is known to play a critical role in mediating inflammatory responses 11, we further analyzed this pathway via PPI network analysis. The network analysis predicted a potential interaction and a high clustering coefficient among *Nlrp3, Caspase1* and *Il1b*, three critical components of NLRP3 inflammasome signaling. AOS3 treatment led to a significant decrease in *Nlrp3, Caspase* and* Il1b* gene overexpression in the affected joints (Figure 4H). Since NLRP3 inflammasome plays a pivotal role in mediating gouty arthritis inflammation and joint pain, the RNA-Seq results indicate that NLRP3 inflammasome may be potential molecular target of AOS3' therapeutic effect on gouty arthritis.

The NLRP3 inflammasome is a complex of proteins responsible for the cleavage of pro-IL-1β into active mature IL-1β that plays an important role in mediating pain and inflammation in gouty arthritis 11. We therefore continued to validate whether AOS3 may interfere with NLRP3 inflammasome signaling by protein assay. Western blotting showed that the protein expression of 4 major components in NLRP3 inflammasome, namely NLRP3, cleaved Caspase-1, ASC and cleaved IL-1β, were all significantly up-regulated in the inflamed ankle joint tissues of gouty arthritis model mice 24 h after MSU injection (Figure 4I-K), indicating NLRP3 inflammasome activation. Importantly, AOS3 treatment significantly reduced NLRP3, cleaved Caspase-1, ASC and cleaved IL-1β overexpression in ankle joints of model mice (Figure 4I-K). This result is consistent with our prior RNA-Seq screening results. Taken together, the above data indicates that AOS3 treatment inhibits NLRP3 inflammasome activation in the inflamed ankle joint of gouty arthritis model mice.

### AOS3 attenuated MSU-induced ROS overproduction and inflammation

**Figure 5:**
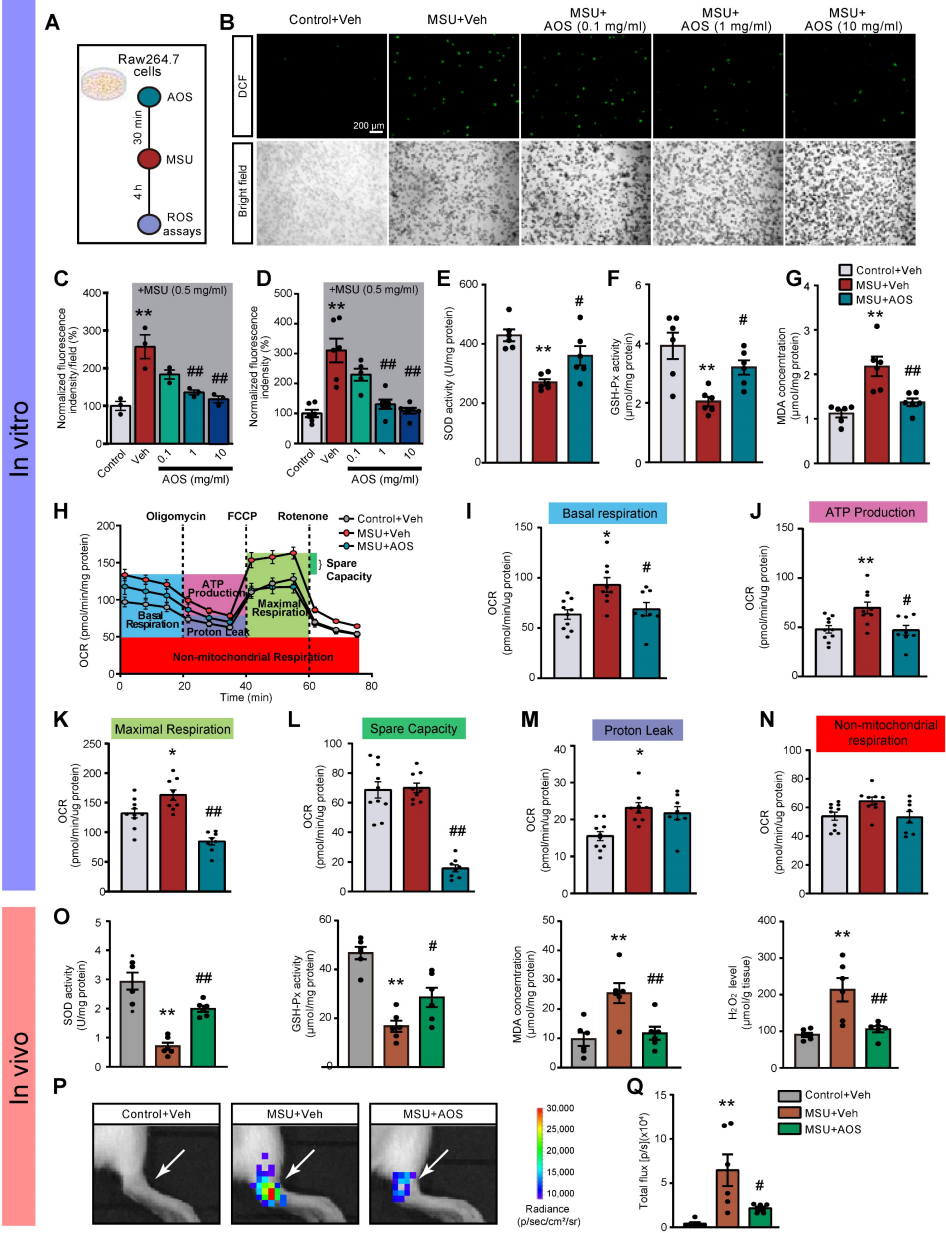
** AOS3 reduces MSU-induced ROS production and improves mitochondrial bioenergetics dysfunction.** (A) Schematic *in vitro* experiments protocol using murine macrophage cell line RAW264.7 cells. (B) Representative photographs showing cellular oxidative stress indicated by DCF fluorescence in control condition and stimulated by MSU (0.5 mg/ml) in RAW264.7 cells captured with a fluorescence microscope. Upper panel: DCF fluorescence. Lower panel: bright field (BF) image. Cells were treated with vehicle (PBS) or AOS3 (0.1, 1 or 10 mg/ml) 30 min before MSU application. Scale bar indicates 200 μm. (C) Summary of DCF fluorescence intensity in Control, MSU+Veh and MSU groups treated with different dosages of AOS (0.1, 1 or 10 mg/ml) as shown in panel B. The control group value was taken as 100% and all other groups were normalized thereafter. n=3 tests/group. (D) Summary of DCF fluorescence intensity in Control, MSU+Veh and MSU groups treated with different dosages of AOS (0.1, 1 or 10 mg/ml) determined by the microplate reader. n=5-6 tests/group. (E-G) Summary of determinations of SOD activities (E), GSH-Px activities (F), and MDA concentrations (G) in Raw264.7 cells treated with vehicle or AOS (1 mg/ml). n=5-6 tests/group. (H) Overlaid time courses showing the determination of OCR in Raw264.7 cells of Control+Veh, MSU+Veh and MSU+AOS groups. Different color-labeled region denotes corresponding OCR parameters. (I-N) Analysis of parameters involved in mitochondrial bioenergetics, including basal respiration (I), ATP production (J), maximal respiration (K), spare respiratory capacity (L) proton leak (M), and non-mitochondrial respiration (N). n=8-10 tests/group. (O) Summarized data showing *in vivo* studies determining SOD activity, GSH-Px activity, and MDA content as well as H_2_O_2_ level in ankle joint tissues 24 h after vehicle/MSU injection. (P) *In vivo* imaging of ROS levels of the ankle joints by means of L-012 in live animals. (Q) Summary of total chemiluminescent fluxes of L-012 in the ankle joints. n=6 mice/group. ^*^p<0.05, ^**^p<0.01 vs. Control+Veh. ^#^p<0.05, ^##^p<0.01vs. MSU+Veh group. One-way ANOVA followed by Tukey's post hoc test was used.

We continued to explore how AOS3 might attenuated NLRP3 inflammasome signaling activation in gouty arthritis. Oxidative stress plays a critical role in mediating NLRP3 inflammasome activation 17, 18. We and others have demonstrated that ROS are excessively generated in the inflamed tissues of gouty arthritis model mice and make important contributions to NLRP3 inflammasome activation 12, 14, 42. Therefore, we aimed at examining the potential effects of AOS3 on oxidative stress in gouty arthritis condition. We first started by *in vitro* testing the effect of AOS3 on ROS production triggered by MSU in a murine macrophage cell line RAW264.7 cells (Figure 5A). The results showed that ROS production was significantly increased in RAW264.7 cells after incubation with MSU (0.5 mg/ml) as measured by DCF fluorescence via fluorescence microscope. The pretreatment with AOS3 dose-dependently reduced ROS overproduction in cells exposed to MSU (Figure 5B&C). To make a further quantitative analysis, we utilized a microplate reader to further quantify DCF fluorescence. Similarly, AOS3 treatment dose-dependently reduced ROS overproduction in cells exposed to MSU (Figure 5D). Furthermore, MSU incubation significantly decreased the activities of antioxidant enzyme, including superoxide dismutase (SOD) and GSH peroxidase (GSH-Px) and further triggered MDA overproduction in RAW264.7 cells (Figure 5E-G). AOS3 treatment significantly reversed the effect of MSU on these oxidative stress markers and products (Figure 5E-G). We then isolated and cultured bone marrow-derived macrophages (BMDMs) from mice and tested the effects of AOS3 on the BMDMs (Figure S5A). Results showed that MSU (0.5 mg/ml) incubation triggered an obvious increase in DCF fluorescence in BMDMs monitored via fluorescence microscope, which was an indication of ROS generation. AOS (1 mg/ml) treatment significantly reduced the increased DCF fluorescence in BMDMs caused by MSU challenge (Figure S5B&C). Quantification of DCF fluorescence using microplate reader showed similar results (Figure S5D). These data demonstrates that AOS3 is able to reduce ROS overproduction induced by MSU in both RAW264.7 cells and primary macrophages *in vitro*.

The malfunction of mitochondrial bioenergetics can end up in an overproduction of mitochondrial ROS, which are major constituents of total cellular ROS generation 43. We hypothesize that AOS3 may inhibit oxidative stress via improving mitochondrial bioenergetics impairments. We tested this hypothesis with Seahorse Bioscience XF96 analyzer that permits analysis of real-time changes in oxygen consumption rate (OCR), a surrogate of mitochondrial respiration, in RAW264.7 cells (Figure 5H). We observed that MSU treatment induced significant impairments in basal respiration, maximal respiration, protein leak and ATP production in RAW264.7 cells. AOS3 treatment effectively reversed mitochondrial bioenergetics dysfunctions in basal respiration, ATP production, maximal respiration and spare respiratory capacity (Figure 5I-N).

Next, we checked the effect of AOS3 on oxidative stress status in gouty arthritis model mice *in vivo*. MSU injection triggered a significant reduction in SOD oxidase activity and GSH-Px activity and further increased productions of MDA and H_2_O_2_ in ankle joint tissues of gout model mice (Figure 5O). AOS3 treatment restored the endogenous antioxidative capacities through replenishing GSH-Px and SOD activities (Figure 5O). AOS3 also reduced the overproduction of MDA and H_2_O_2_ (Figure 5O). We then applied a noninvasive imaging technique to further monitor ROS status *in vivo* in ankle joints of living mice with chemiluminescent probe L-012 44, 45. As shown in Figure 5P&Q, MSU+Veh group mice showed obviously increased L-012 chemiluminescent signals in ankle joints than Control+Veh group mice, demonstrating the existence of excessive oxidative stress in ankle joints. MSU+AOS group showed significantly reduced L-012 signal in ankle joints *vs*. MSU+Veh group (Figure 5P&Q). These above results indicate that AOS3 treatment can produce antioxidative effects in the inflamed ankle joints of gout model mice.

### AOS3 activates Nrf2-mediated antioxidative signaling that contributes to ameliorative effects on pain and inflammation of gouty arthritis model mice

**Figure 6:**
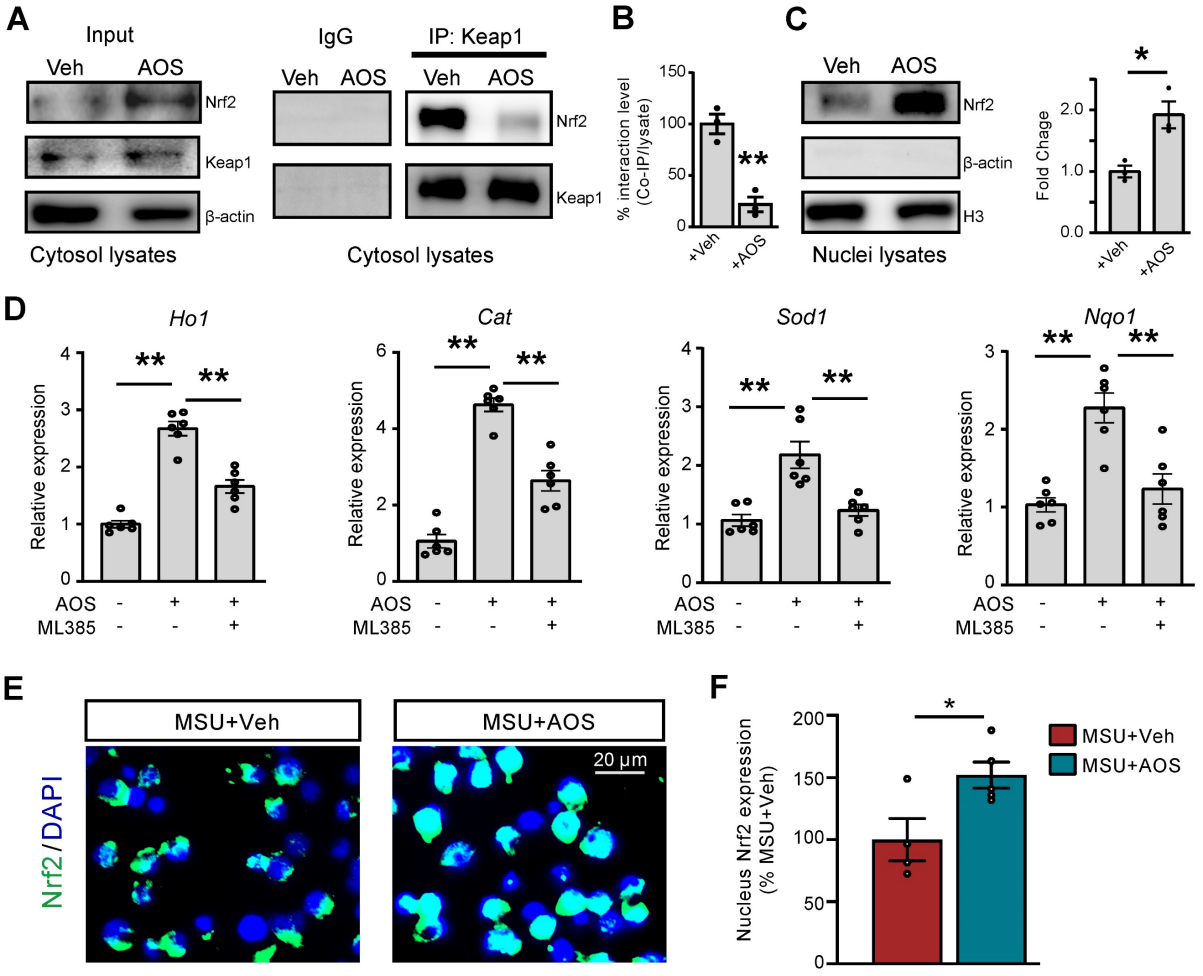
** AOS3 activates Nrf2-related antioxidant signaling cascade *in vitro*.** (A) Representative Co-IP assay showing Keap1-bound Nrf2 expression in cytosol lysates of Raw264.7 cells treated with vehicle or AOS3 (1 mg/ml). IgG was used as a negative control. (B) Summary of Keap1-Nrf2 interaction level determined by Co-IP assay as in panel A. +Veh group was normalized as 100%. n=3 tests/group. (C) Nrf2 protein in nuclear fraction lysates. (D) Gene expression of *Ho1*, *Cat*, *Sod1* and *Nqo1* in RAW264.7 cells with vehicle, AOS3 or AOS3+ML385 treatment. n=6 tests/group. (E) Representative immunocytochemical images of subcellular localization of Nrf2 in Raw264.7 cells treated with vehicle or AOS in the presence of MSU. Blue: DAPI. Green: Nrf2. (F) Summarized data shows quantification of Nrf2 in the nucleus. n=4-5 tests/group. ^**^p<0.01, ^*^p<0.05. Unpaired Student's *t* test was used for panels B, C&F. One-way ANOVA followed by Tukey's post hoc test was used for panel D.

The transcription factor Nrf2-associated antioxidative signaling constitutes a primary endogenous defensive mechanism against oxidative stress 46-49. In order to further explore the upstream mechanisms leading to AOS-mediated antioxidative effect, we examined whether AOS3 might activate Nrf2 signaling in gouty arthritis condition. We first tested the effect of AOS3 on Nrf2 in RAW264.7 cells. Co-immunoprecipitation (Co-IP) experiments using the cytosol fraction lysate showed that Nrf2-Keap1 association was significantly disrupted by AOS3 treatment, resulting in dissociation of Nrf2 from Keap1-bounded complex. In contrast, Keap1 protein expression per se remained unchanged in cytosol (Figure 6A&B). The dissociated Nrf2 protein further translocate to the nuclei, which was illustrated by the significant increase in Nrf2 protein expression in nuclear fraction lysates of AOS-treated RAW264.7 cells (Figure 6C). As a result, mRNA expressions of certain Nrf2-antioxidative signaling-related genes, including *Ho1*, *Cat*, *Sod1* and *Nqo1*, were all significantly up-regulated in RAW264.7 cells, whereas pretreatment with Nrf2 specific inhibitor ML385 reversed the up-regulation of these genes by AOS3 (Figure 6D). Immunofluorescence further confirmed that AOS treatment significantly promoted the nucleus Nrf2 expression compared with vehicle treatment in RAW264.7 cells incubated with MSU (Figure 6E&F). Thus, these data demonstrates that AOS3 can activate Nrf2-mediated antioxidative signaling in RAW264.7 cells challenged with MSU.

**Figure 7:**
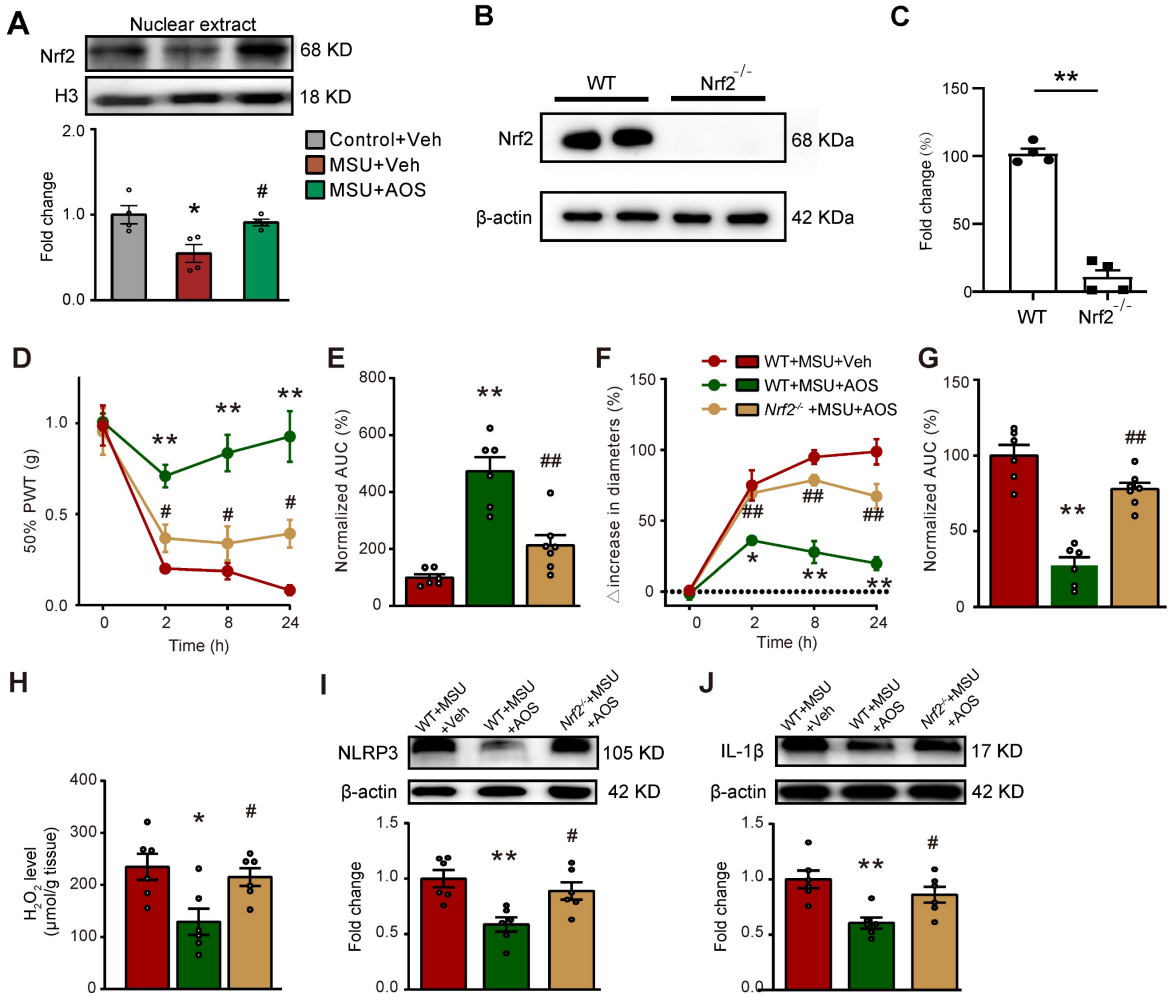
** AOS3 ameliorates mechanical pain and ankle edema of gouty arthritis model mice via Nrf2-dependent mechanism.** (A) The expression of Nrf2 in nuclear fraction lysates of ankle joint tissues of Control+Veh, MSU+Veh and MSU+AOS group of mice determined by Western blotting. ^*^p<0.05 vs. Control+Veh group. ^#^p<0.05 vs. MSU+Veh group. (B) Western blotting examining Nrf2 expression in ankle joint tissues of wildtype (WT) and *Nrf2*^-/-^ mice. **p<0.01 vs. WT group. (C) Overlaid time courses showing 50% PWT changes in 3 groups of mice. AOS3 (200 mg/kg, i.p.) or vehicle (PBS) was administered at time points as in Figure 1A. (D) Normalized AUC analysis of curves in panel C. (E) Overlaid time courses showing ankle joint diameter changes in 3 groups of mice. (F) Normalized AUC analysis of curves in panel E. (G) Biochemical assays of H_2_O_2_ level in WT+MSU+Veh, WT+MSU+AOS and *Nrf2*^-/-^+MSU+AOS groups of mice. (H&I) Western blots examining NLRP3 and IL-1β expressions in ankle joint tissues. ^*^p<0.05, ^**^p<0.01 vs. WT+MSU+Veh group. ^#^p<0.05, ^##^p<0.01 *vs.* WT+ MSU+AOS group. n = 4-6 mice/group. Two-way ANOVA followed by Tukey's post hoc test was used for panel D&F. Unpaired Student's *t* test was used for panel C. One-way ANOVA followed by Tukey's post hoc test was used for others.

To follow up with the above *in vitro* results, we continued to test whether AOS3 treatment could promote Nrf2 signaling in the inflamed ankle joint tissues of gouty arthritis model mice *in vivo*. We extracted the nucleus fraction of ankle joint tissues from gouty arthritis model mice and examined Nrf2 expression. We found AOS3 treatment could significantly promote Nrf2 expression in the nucleus extractions of ankle joints from gouty arthritis model mice (Figure 7A). In order to further investigate whether Nrf2-mediated antioxidative signaling contributes to AOS's effects on gouty arthritis mice, we employed Cas9/gRNA technique to generate *Nrf2* gene deficient (*Nrf2*^-/-^) mice by deleting the exon 2-5 encoding *Nrf2* gene. The efficiency of *Nrf2* gene deficiency was evaluated by Western blot, which showed that Nrf2 protein expression in ankle joint was almost totally eliminated in *Nrf2*^-/-^ mice compared with wild type (WT) mice (Figure 7B&C). As shown in Figure 7D, no significant differences in baseline levels of PWT between *Nrf2*^-/-^ and WT mice were observed, demonstrating no deficiency in basal mechanical pain perception in *Nrf2*^-/-^ mice. The administration of AOS3 (200 mg/kg) significantly reversed mechanical allodynia and joint swelling in WT mice upon MSU-injection (Figure 7D-G). In contrast, AOS3 failed to exert therapeutic effect on mechanical allodynia and edema in *Nrf2*^-/-^ mice (Figure 7D-G). Corroborating with the results obtained by behavioral assay, AOS3-induced antioxidative effects, including the reduction of ROS product H_2_O_2_ in ankle joint tissues of gout model mice, was abolished in *Nrf2*^-/-^ mice (Figure 7H). We further checked NLRP3 inflammasome signaling. Results showed that *Nrf2* deficiency near completely abolished AOS3's attenuating effect on NLRP3 and IL-1β overexpression in ankle joints of gouty arthritis model mice (Figure 7I&J). Thus, these above observations indicate that AOS3 produce ameliorative effects on gouty arthritis model mice through triggering Nrf2-mediated antioxidative signaling in the inflamed ankle joints.

### AOS3 attenuates inflammatory cell infiltration in ankle joints of gouty arthritis model mice

**Figure 8:**
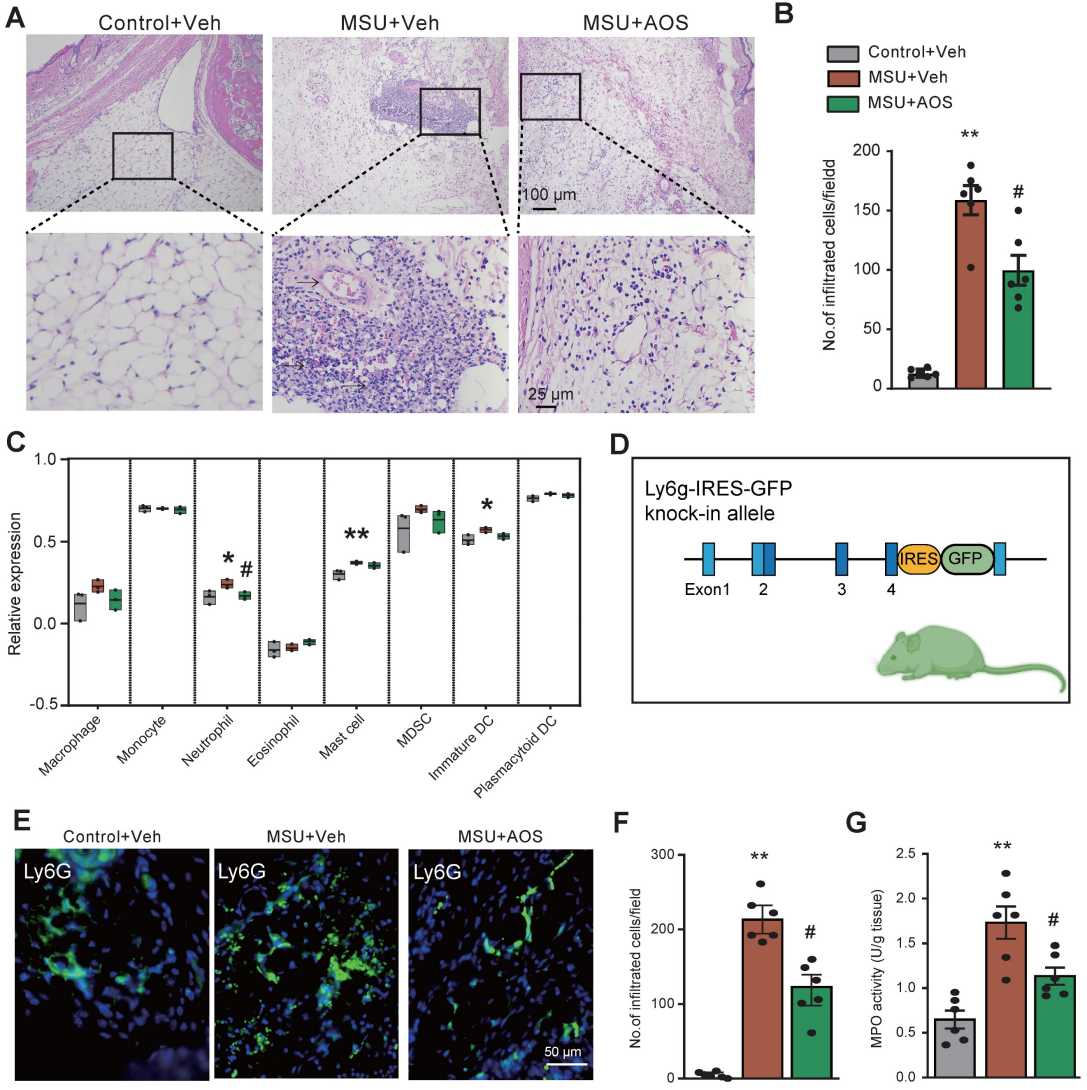
** AOS3 treatment reduces inflammatory cell infiltrations in ankle joints of gouty arthritis model mice.** (A) Representative pictures showing H&E staining of ankle tissues from Control+Veh, MSU+Veh and MSU+AOS groups of mice. Bottom panel shows the enlarged field depicted by black boxes on the top. (B) Summary of the number of inflammatory cells infiltrated per observation field. (C) The amount of cell abundance in ankle joint calculated by ssGSEA score of each cluster based on marker genes using RNA-Seq dataset in Figure 4. (D) The strategy of generating Ly6G-IRES-GFP knock-in mouse line. (E) Representative pictures showing fluorescence of Ly6G-GFP^+^ neutrophils in ankle tissue sections of Ly6G-IRES-GFP knock-in mouse from Control+Veh, MSU+Veh and MSU+AOS group. (F) Summary of the number of Ly6g-GFP^+^ cells per observation field. Scale bar indicated 50 μm. (G) Summary data showing MPO activity assay. n=6 mice/group. ^*^p<0.01, ^**^p<0.01 vs. Control+Veh. ^#^p<0.05, ^##^p<0.01 vs. MSU+Veh group. One-way ANOVA followed by Tukey's post hoc test was used for panels B, F&G.

As illustrated by H&E staining in Figure 8A-B, there were a large amount of inflammatory cells accumulated in the periarticular tissues of ankle joints of gouty arthritis model mice, which was ameliorated by the application of AOS3. To further explore the specific types of immune cells that AOS may affect, we utilized ssGSVA package to further analyze our RNA-Seq dataset (as shown in Figure 4). The bioinformatics analysis identified a notable infiltration of neutrophils, mast cells, as well as immature dendritic cells (DCs) in ankle joints of MSU+Veh group vs. control group (Figure 8C). Notably, treatment with AOS3 significantly decreased the extent of neutrophils recruitment to the inflamed ankle joints (Figure 8C). In order to visualize the neutrophils more directly, we generated a Ly6G-IRES-GFP knock-in mouse line using Cas9-associated guide RNA (gRNA) technique (Figure 8D). The mouse has GFP inserted into Ly6G (a marker for neutrophil) allele, thus enabling the identification of neutrophils via the GFP signal 14, 16. With the aid of this mouse line, we found that AOS3 treatment could significantly attenuate neutrophil infiltration in periarticular tissues of gouty arthritis model mice (Figure 8E&F, MSU+AOS *vs.* MSU+Veh). Myeloperoxidase (MPO) activity assay was further applied to quantify the amount of neutrophils accumulated in the ankle joint. Results showed that MPO activity was significantly up-regulated in ankle joints of the model mice, indicating neutrophil infiltrations. AOS3 treatment reduced the up-regulated MPO activity in ankle joints of model mice (Figure 8G). These results indicate that AOS3 treatment is capable of attenuating excessive neutrophil infiltration in ankle joints of gouty arthritis model mice.

We further checked the expressions of some pro-inflammatory and anti-inflammatory genes. Gene expressions of certain pro-inflammatory cytokines, including *Il1b, Tnfa*, *Il6*, *Cxcl1* and *Cxcl2* were significantly upregulated in ankle joint tissues of gout model mice (Table 1). AOS3 treatment significantly attenuated gene overexpression of *Il1b*, *Tnfa*, *Il6* and *Cxcl2* genes, whereas it had no obvious effects on *Il4* and *Il10* expression, two anti-inflammatory genes (Table 1).

### AOS3 attenuates enhanced TRPV1 channel function in sensory neurons and reduces neuropeptide overproduction in ankle joint tissues

**Figure 9:**
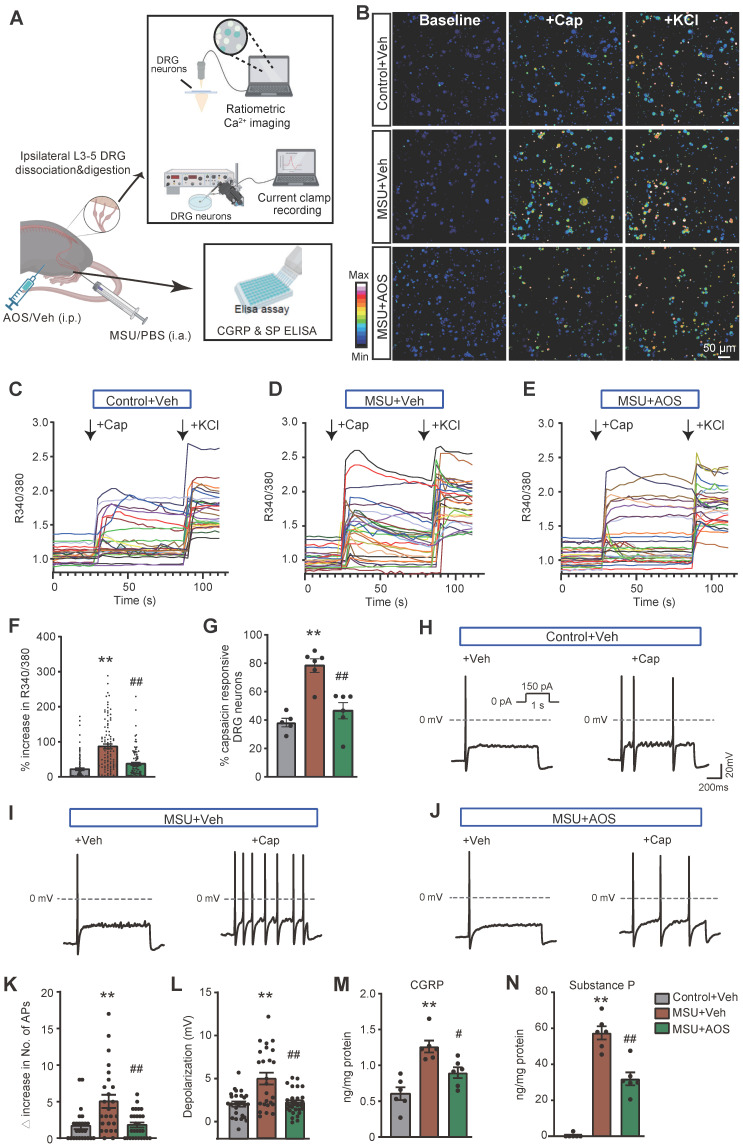
** AOS3 reduces the enhanced TRPV1 channel activity in DRG neurons and reduced neuropeptide release in the inflamed ankle joints of gouty arthritis model mice** (A) Cartoon showing the procedure for Ca^2+^ imaging and current clamp on dissociated DRG neurons and ELISA test on ankle joint tissues. (B) Pseudo-color images from Fura-2-based ratiometric Ca^2+^ imaging showing the Ca^2+^ responses in DRG neurons in response to TRPV1 specific agonist capsaicin (Cap, 300 nM) in the Control+Veh, MSU+Veh, and MSU+AOS groups. KCl (50 mM) was perfused at the end to determine all active DRG neurons. Scale bar indicates 50 μm. (C-E) Overlaid Ca^2+^ imaging traces of DRG neurons isolated from three groups. DRG neurons were perfused with capsaicin, followed with KCl. The arrows indicate the time point of drug application. (F) Summary of Δ increase in peak Ca^2+^ transients triggered by capsaicin of 3 groups. n=100 neurons/group. (G) Summarized percentage of capsaicin responsive DRG neurons in each observation field from 3 groups of mice. n=5-6 tests/group. (H-J) Representative action potentials (APs) elicited by application of capsaicin (100 nM) in 3 groups recorded by current clamp mode. (K) Summary of the Δ increase in the No. of APs elicited by capsaicin (100 nM) in DRG neurons of 3 groups. 25, 25 and 28 neurons were included in Control+Veh, MSU+Veh, and MSU+AOS groups, respectively. (L) Summary of membrane potential depolarization triggered by applying capsaicin (100 nM) in DRG neurons of 3 groups. 25, 25 and 28 neurons were included in Control+Veh, MSU+Veh, and MSU+AOS groups, respectively. (M&N) ELISA test of CGRP and SP levels in ankle joint tissues. n = 5-6 tests/group. ^**^p<0.01 vs. Control+Veh. ^#^p<0.05, ^##^p<0.01vs. MSU+Veh group. One-way ANOVA followed by Tukey's post hoc test was used for analysis.

Our group and others recently found that pain-sensing TRPV1 channel expression and activity were significantly increased in DRG neurons innervating the hind limbs of gouty arthritis model mice 12, 14, 50. We further demonstrated pharmacological inhibition of NLRP3 inflammasome significantly reduced TRPV1 overexpression in DRG neurons of gouty arthritis model mice, indicating an important role of NLRP3 inflammasome in modulating TRPV1 expression 14. Since we found AOS could target against NLRP3 inflammasome in this study, we next wanted to examine whether AOS treatment would reduce the enhanced TRPV1 channel activity in DRG neurons of gouty arthritis model mice. Ipsilateral L3-L5 DRG innervating the hind limb was dissociated, cultured and then subject to live cell Ca^2+^ imaging (Figure 9A). Applying TRPV1 specific agonist capsaicin (300 nM) elicited significantly higher Ca^2+^ transients in DRG neurons isolated from MSU group than control group. AOS3 treatment significantly reduced the enhanced Ca^2+^ response triggered by capsaicin (Figure 9B-F). Furthermore, the % of capsaicin responsive neurons (among all KCl responsive neurons) was significantly higher in MSU group than control group (Figure 9G). AOS3 treatment significantly reduced the increased % of neurons responding to capsaicin (Figure 9G). In addition, acute bath application of AOS3 did not interfere with capsaicin-induced Ca^2+^ responses in DRG neurons, ruling out the possibility of direct inhibitory effect of AOS on TRPV1 channel (Figure S6).

We further tested the effects of AOS treatment on excitability of DRG neurons elicited by activating TRPV1 channel via capsaicin. Ipsilateral L3-L5 DRG innervating the hind limb was dissociated, cultured and then subject to current clamp recording (Figure 9A). We selected small-sized DRG (with Cm < 42 pF) for patch clamp recording as in our recent studies since a majority of these neurons are nociceptive 37, 51. Action potentials were elicited by injecting 150 pA current as shown in Figure 9H inset. It was apparent that applying capsaicin (100 nM) triggered more action potentials (APs) in DRG neurons from MSU+Veh group than control group (Figure 9H-K). The hyperexcitability in DRG neurons triggered by capsaicin was significantly reduced by AOS treatment (Figure 9H-K). Moreover, capsaicin triggered more membrane potential depolarization in DRG neurons from MSU+Veh group vs. control+Veh group, which was significantly reduced in MSU+AOS group (Figure 9L).

TRPV1 is predominantly expressed in peptidergic nociceptors, the activation of which contribute to neurogenic inflammation via the release of neuropeptide, including CGRP and SP. These two neuropeptides contribute to vessel dilation and extravasation, two processes facilitating inflammatory cell infiltration 52. We then examined the effects of AOS3 on CGRP and SP in the inflamed ankle joints of gouty arthritis model mice. ELISA showed that CGRP and SP levels were both significantly increased, whereas AOS3 treatment resulted in significantly less CGRP and SP levels (Figure 9M&N). These above findings demonstrate that AOS treatment can attenuate the enhanced TRPV1 channel activity in DRG neurons and further constrained neuropeptide release in gouty arthritis model mice.

## Discussion

Here in this study, we examined the potential effects of AOSs with specific Dp on joint pain and inflammation of a mouse model of gouty arthritis. We found that AOS2&3 produced the most obvious analgesic and anti-inflammatory effects. We chose AOS3 for further mechanism study. AOS3 dose-dependently relieved signs of gouty arthritis and reversed gait impairments in model mice. AOS3 inhibited NLRP3 inflammasome signaling activation and pro-inflammatory cytokine up-regulation in ankle joints of model mice. AOS3 reduced MSU-induced oxidative stress both *in vivo* and *in vitro* and reversed the impaired mitochondrial bioenergetics. AOS3 activated Nrf2 anti-oxidant signaling to promote antioxidant gene expressions. AOS3's therapeutic effects on joint pain, inflammation and oxidative stress of model mice were all abolished in *Nrf2*^-/-^ mice. AOS3 treatment further reduced the enhancement in pain-sensing TRPV1 channel function in DRG neurons and neuropeptide overproduction in ankle joints of model mice. These findings indicate that AOS ameliorates gouty arthritis pain and inflammation via activating Nrf2-dependent antioxidant signaling, resulting in the suppression of ROS-mediated NLRP3 inflammasome activation and TRPV1 channel enhancement.

Currently, a majority of studies are performed using undefined AOSs mixtures with ununiformed Dp, thus making it hard to interpret the exact contributions of each AOS polymer 22. Studies have shown that AOSs with different Dp exerted distinct biological activities. For example, AOSs-induced antitumor effects have been explored among 4 AOSs with distinct Dp (Dp2-5) in osteosarcoma cells. However, only AOS5 (Dp=5) exhibited antitumor effect 53. Therefore, in this study, three AOSs with specific Dps were evaluated and the therapeutic potentials were compared. Results showed that AOS2, AOS3 and AOS4 all exhibited anti-inflammatory property in gouty arthritis model mice. However, AOS2 and AOS3 exhibited more obvious analgesic effect in gout model mice compared with AOS4. AOS2 or AOS3 could achieve anti-inflammation and analgesia to similar extent with indomethacin in gouty arthritis model mice. These findings unraveled that AOSs with different Dp may exert distinct analgesic effect in a mouse model of gouty arthritis. Therefore, Dp is a factor that should be taken into account when evaluating AOS's anti-gouty arthritis activity in the future.

To identify potential molecular target of AOSs' therapeutic effects on gouty arthritis, we performed an unbiased genome wide RNA-Seq to screen signaling pathways that AOS3 may affect. RNA-Seq revealed that NOD-like receptor signaling pathway was remarkably activated during gouty arthritis, whereas AOS3 treatment significantly reduced the activation. PPI network analysis further identified that *Nlrp3* and *Il1b* gene expression were predominantly affected by AOS3's treatment. It is known that NLRP3 inflammasome plays a critical role in mediating gouty arthritis pain and inflammation 11, 54. The engulfment of MSU by tissue resident macrophages triggers the activation of NLRP3 inflammasome, which produces IL-1β 9. The released IL-1β serves as a primary pro-inflammatory cytokine in both inflammation and pain of gouty arthritis. Our protein assay validated the result from RNA-Seq by showing that AOS3 treatment significantly reduced the overexpression of signaling molecules involved in NLRP3 inflammasome pathway, including NLRP3, Caspase-1, ASC and IL-1β. Therefore, the inhibition of NLRP3 inflammasome may contribute to AOS3's therapeutic effect on gouty arthritis. Some prior studies revealed that the mechanisms involved in AOS3's anti-inflammatory effects include inhibition of TLR4, p38MAPK or NF-κB-mediated signaling or modulation of gut microbiota, etc. (see review 22). Here, we further propose that AOS3 can inhibit NLRP3 inflammasome activation. As far as we know, this is the first study revealing that AOS possess the capability of inhibiting NLRP3 inflammasome signaling during gouty arthritis, which provides a novel mechanism to explain AOS-induced anti-inflammation.

ROS serves as a critical triggering factor for NLRP3 inflammasome activation 55. Furthermore, ROS is indispensable for MSU-induced NLRP3 inflammasome activation 56. There are studies showing that ROS are generated from the inflamed joint tissues of gouty arthritis model animals 13, 57, 58. Antioxidants are able to reduce NLRP3 inflammasome activation via decreasing ROS overproduction in the inflamed joint tissues of gouty arthritis model animals 12. These studies suggest that ROS are key factors contributing to NLRP3 inflammasome activation in gouty arthritis condition. Studies have shown that AOSs possess antioxidant capability of reducing ROS overproduction and up-regulating the antioxidant system 59-61. Therefore, to further understand the mechanism underlying how AOSs may inhibit NLRP3 inflammasome activation, we examined AOS3's effect on ROS production in gouty arthritis model mice. We found that AOS3 can reduce ROS overproduction both in RAW264.7 cells and primary macrophages treated with MSU *in vitro* and in ankle joints of gouty arthritis mice *in vivo*. This result suggests that AOS3 can exert anti-oxidative effect in gout model mice, which may contribute to its alleviation of gout pain and inflammation.

We continued to investigate how AOS3 could exert anti-oxidative effect in gouty arthritis condition. It is known that Nrf2 antioxidant signaling constitutes an important endogenous machinery to counteract oxidative stress via promoting antioxidant gene expression. Prior studies revealed that AOS could activate Nrf2 signaling 59, 62, 63. But it remains largely unknown how exactly AOS activates Nrf2 signaling. Here, we showed by Co-IP experiments that AOS3 promoted the dissociation of Nrf2 from Nrf2-Keap1 complex in the cytosol and promoted its nuclear translocation in RAW264.7 cells. As a consequence, AOS3 up-regulated Nrf2-related antioxidant gene expression. Moreover, AOS-induced gene up-regulation was blocked by Nrf2 specific blocker ML385, further demonstrating Nrf2 was the molecular target of AOS3. These *in vitro* findings were corroborated by the *in vivo* findings that AOS3 treatment promoted Nrf2 nuclear accumulation in ankle joint tissues of gout model mice. To further confirm whether AOS3 targets Nrf2 signaling to exert therapeutic effects *in vivo*, we made use of *Nrf2*^-/-^ mouse strain. We first confirmed that Nrf2 protein expression was completely eliminated in *Nrf2*^-/-^ mice. In *Nrf2*^-/-^ mice, AOS3 failed to ameliorate mechanical allodynia and joint edema elicited by MSU injection. Moreover, AOS3-induced antioxidant effect and attenuation of NLRP3 inflammasome in joint tissues were both abolished in *Nrf2*^-/-^ mice. These *in vitro* and *in vivo* results in together indicate that AOS3 produces ameliorative effects on gouty arthritis model mice via promoting Nrf2-mediated antioxidant signaling.

When MSU crystals deposit in the joint, local resident macrophages are the first type of cells responding to MSU 9. Macrophages execute phagocytosis to engulf MSU crystals, which results in the activation of NLRP3 inflammasome signaling and the generation of pro-inflammatory cytokines, including IL-1β, IL-18, IL-6, TNF-α and CXCL5 37, 64, 65. These cytokines are associated with increased blood vessel permeability, neutrophil recruitment, and the subsequent inflammation cascade. So macrophages serve as the primary cells to initiate and drive the early inflammatory response to MSU deposition 10. In our study, we found that AOS3 treatment can reduce MSU-induced ROS production in both RAW264.7 cells and native macrophages. Furthermore, AOS3 can activate Nrf2 signaling in RAW264.7 cells to promote antioxidant gene expression. These results in together indicate that AOS3 can act on macrophages to dampen their activation by MSU, resulting in less production of pro-inflammatory cytokines from macrophages. The reduced pro-inflammatory cytokines release may result in less blood vessel permeability and less neutrophil influx as we observed. However, the likelihood that AOS may directly affect neutrophil infiltration mechanism cannot be excluded. For example, fucoidan, a polysaccharide also derived from brown seaweeds, can inhibit leukocyte infiltration by blocking P- and L-selectin, which mediate leukocyte rolling and adhesion along vascular wall 66, 67. Therefore, further detailed mechanism studies are still warranted to clarify this possibility.

We and others all have found that pain-sensing TRPV1 channel expression is significantly up-regulated in both ankle joint tissues and innervating DRG neurons in gouty arthritis model mice 12, 14, 68. The joint pain and inflammation can be reduced by applying TRPV1 specific antagonist in gouty model mice 68, 69. These findings demonstrate an important contribution of TRPV1 channel in gouty arthritis pain and inflammation. The overexpressed nociceptive TRPV1 channel can result in peripheral sensitization that further exacerbates pain and neurogenic inflammation. Recently, our group discovered that TRPV1 channel expression was up-regulated in DRG neurons through oxidative stress/NLRP3 inflammasome-dependent mechanism during gouty arthritis 14, 16. ROS generated in the inflamed tissue can facilitate NLRP3 inflammasome activation and IL-1β production that enhances TRPV1 expression in sensory neurons 14. Pharmacological blocking NLRP3 inflammasome using the selective blocker MCC950 significantly reduced the TRPV1 overexpression in DRG neurons of gouty arthritis model mice 14. Furthermore, our previous study confirmed the *in vivo* effectiveness of MCC950 by showing that NLRP3 inflammasome activation and IL-1β overexpression in ankle joints of gouty arthritis model mice was significantly reduced after MCC950 treatment 14. Thus, these results in all have demonstrated that ROS-mediated NLRP3 inflammasome activation contributes to TRPV1 upregulation in DRG neurons of gouty arthritis model mice. Here in this study, we found that AOS3 treatment could ameliorate the enhanced functional activity of TRPV1 channel in DRG neurons. Furthermore, we did not observe that AOS3 could directly inhibit TRPV1 channel activation in DRG neurons. Therefore, the inhibitory effect of AOS3 on TRPV1 functional enhancement may be largely due to its capability of inhibiting ROS generation and subsequent NLRP3 inflammasome activation in the inflamed ankle joints of gouty arthritis model mice.

Indomethacin is used as a first-line medication for relieving acute gout flares 6. However, indomethacin can cause obvious adverse effects, including peptic ulcer and nephrotoxicity. These adverse effects can become more severe especially among patients with history of peptic ulcer and comorbidities like chronic kidney disease 7, 70. Here, we found that AOS3 can achieve similar efficacy in alleviating joint pain and inflammation in an animal model of acute gouty arthritis. At present, there are still no studies reporting toxicity in AOS as far as we know. Recently, one review paper summarized all the safety studies of AOS and found that AOS had no obvious toxicity and side-effects for different cell lines, mouse models and human patients, which indicated safety of AOS for utilization as food supplements, drug carriers or pharmaceutical ingredients 21. In our study, we did not observe any obvious side effects of AOS, either. Therefore, in terms of safety, AOS may have an advantage over indomethacin in relieving gouty arthritis.

## Conclusions

AOS3 ameliorates gouty arthritis via activating Nrf2-dependent antioxidant signaling, resulting in the suppression of ROS-mediated NLRP3 inflammasome activation and TRPV1 channel functional enhancement. We propose that AOS3 may be a potential option for gouty arthritis treatment.

## Supplementary Material

Supplementary figures and table.

## Figures and Tables

**Table 1:** Summary of pro-inflammatory gene expression changes in ankle joints of gout model mice by AOS3 treatment.

Cytokines	Control+Veh	MSU+Veh	MSU+AOS
*Il1b*	1	5.07±0.26^**^	1.40±0.10**^##^**
*Il6*	1	6.28±0.50^**^	3.31±0.16**^#^**
*Tnfa*	1	3.54±0.31^**^	1.62±0.14**^##^**
*Cxcl1*	1	3.27±0.15^**^	2.41±0.07
*Cxcl2*	1	4.12±0.33^**^	1.58±0.14**^##^**
*Il10*	1	1.42±0.08^*^	1.11±0.02
*Il4*	1	0.97±0.02	1.22±0.06

^*^p<0.05, ^**^p<0.01 vs. Control+Veh group. ^#^p<0.05, ^##^p<0.01 vs. MSU+Veh group.
