# Supplementary material for: Well-defined alginate oligosaccharides ameliorate joint pain and inflammation in a mouse model of gouty arthritis
Source: Theranostics. 2024 May 19;14(8):3082–103. doi: 10.7150/thno.95611 (PMC11155397; doi:10.7150/thno.95611)
Supplement: Supplementary file 1 — Supplementary figures and table. [file thnov14p3082s1.pdf]

Supplementary figure legends

Figure S1 Molecular mass of AOS2, AOS3 and AOS4 determined by negative ion electrospray ionization mass spectrometry (ESI-MS).

Figure S2 Effects of AOS3 treatment on heat hyperalgesia and development at later time points in a mouse model of gouty arthritis. (A) Experimental protocol illustrating the time points for model establishment, AOS3 (200 mg/kg), indomethacin (Indo, 10 mg/kg) or corresponding vehicle injections (i.p.), and thermal pain measurements. (B) Time course showing PWL changes of 4 groups. (C) Protocol for AOS3 (200 mg/kg, i.p.) or vehicle (Veh) treatment on the development at the later time points. (D-E) Effect of AOS3 or Veh on mechanical allodynia (D) and ankle diameter (E) of model mice. n=6 mice/group. *p<0.05, **p<0.01. Two-way ANOVA followed by Tukey's post hoc test was used for statistics.

Figure S3 AOS3 treatment does not affect locomotor activities of mice. (A) Movement traces from open filed test of mice which received vehicle (PBS) or AOS3 treatment (200 mg/kg). (B) Summary of the total travel distance of two groups of mice as in panel A. n=7 mice/group. (C) Motor function was tested through Rotarod assay. n=4 mice/group. NS: no significant difference. Unpaired Student's *t* test was used for statistics.

Figure S4 AOS3 relieves gout arthritis pain when administered in post-treatment manner. (A) Schematic picture showing the time points of model establishment, drug administration and behavioral assay. (B) Time course effect of AOS on mechanical allodynia of gout arthritis model mice when applied at time points after model establishment. Oral gavage (o.g.) was used for Veh/AOS3 deliver in mice. (C) Summary of AUC analysis of the curves as show in A. n=6 mice/group. **p<0.01 vs. Control+Veh group. $^{##}$p<0.01 vs. MSU+Veh group. Two-way ANOVA followed by Tukey's post hoc test was used for panel B. One-way ANOVA followed by Tukey's post hoc test was used for panel C.

Figure S5 AOS3 reduces MSU-induced ROS overproduction in primary macrophages.

(A) Schematic protocol showing the experiments using mouse bone marrow-derived macrophages (BMDMs) from tibia and femoral bones. (B) Representative pictures of DCF determination of ROS in BMDMs induced by MSU (0.5 mg/ml) incubation. Cells were incubated with AOS3 (AOS, 1mg/ml) or corresponding vehicle (Veh) 30 min before MSU application. Scale bar indicates 200 μm. Upper panel: DCF fluorescence images. Lower panel: bright field images. (C) Normalized mean fluorescence intensity of DCF in panel B. The control group value was taken as 100% and all other groups were normalized thereafter. n=3 tests/group. (D) Summary of DCF fluorescence intensity of 3 groups determined by the microplate reader. n=6 tests/group. *$p<0.05$, **$p<0.01$. One-way ANOVA followed by Tukey's post hoc test was used for statistics.

Figure S6 Bath application of AOS3 does not interfere with capsaicin-induced Ca^{2+} response in DRG neurons. (A) Summary of % capsaicin responsive DRG neurons in the presence of vehicle or AOS3. 300 nM capsaicin was used to activate TRPV1 channel. AOS3 (1 mg/ml) or vehicle was co-applied with capsaicin. n=6 tests/group. (B) Summary of Δ change in R340/380 elicited by capsaicin (300 nM) in the presence of vehicle or AOS (1 mg/ml). n=100 neurons/group. Unpaired Student's *t* test was used for statistics.

Table S1 Primer design for qPCR test in this study.

Gene Symbol &Gene ID	Primer sequence (5' to 3')	Amplicon size (bp)
β-actin	F:GTGCTATGTTGCTCTAGACTTCG	174
11461	R:ATGCCACAGGATTCCATACC	
Il1β	F:5'-CAACTGTTCCTGAACTCAACTG	281
16176	R:5'-GAAGGAAAAGAAGGTGCTCATG	
Tnfα	F:5'-ATGTCTCAGCCTCTTCTCATTC-3'	179
21926	R:5'-GCTTGTCACTCGAATTTTGAGA-3'	
Cxcl1	F:5'-AAGAATGGTCGCGAGGCTTG-3'	121
14825	R:5'-AGGTGCCATCAGAGCAGTCT-3'	
Cxcl2	F:5'-GGTTGACTTCAAGAACATCCAG-3'	84
114105	R:5'-TTGAGAGTGGCTATGACTTCTG-3'	
Il6	F:5'-CTCCCAACAGACCTGTCTATAC-3'	97
16193	R:5'-CCATTGCACAACTCTTTTCTCA-3'	
Il10	F:5'-TTCTTTCAAACAAAGGACCAGC-3'	81
16153	R:5'-GCAACCCAAGTAACCCTTAAAG-3'	
Il4	F:5'-AGTTGTCATCCTGCTCTTCTTTCTC-3'	119
16189	R:5'-ATGGCGTCCCTTCTCCTGTG-3'	
Ho1	F:5'-AAGACCGCCTTCCTGCTCAAC-3'	106
24451	R:5'-TCTGACGAAGTGACGCCATCTG-3'	
Sod1	F:5'-GTCGGCTTCTCGTCTTGCTCTC-3'	91
20655	R:5'-GCACGCACACCGCTTTCATC-3'	
Cat	F:5'-GTCCCTGCTGTCTCACGTTCC-3'	119
12359	R:5'-CCGCTGCTCCTTCCACTGC-3'	
Nqo1	F:5'-TCCTGCGTTTCTGTGGCTTCC-3'	80
18104	R:5'-CATGCGGGCATCTGGTGGAG-3'	

Figure S1

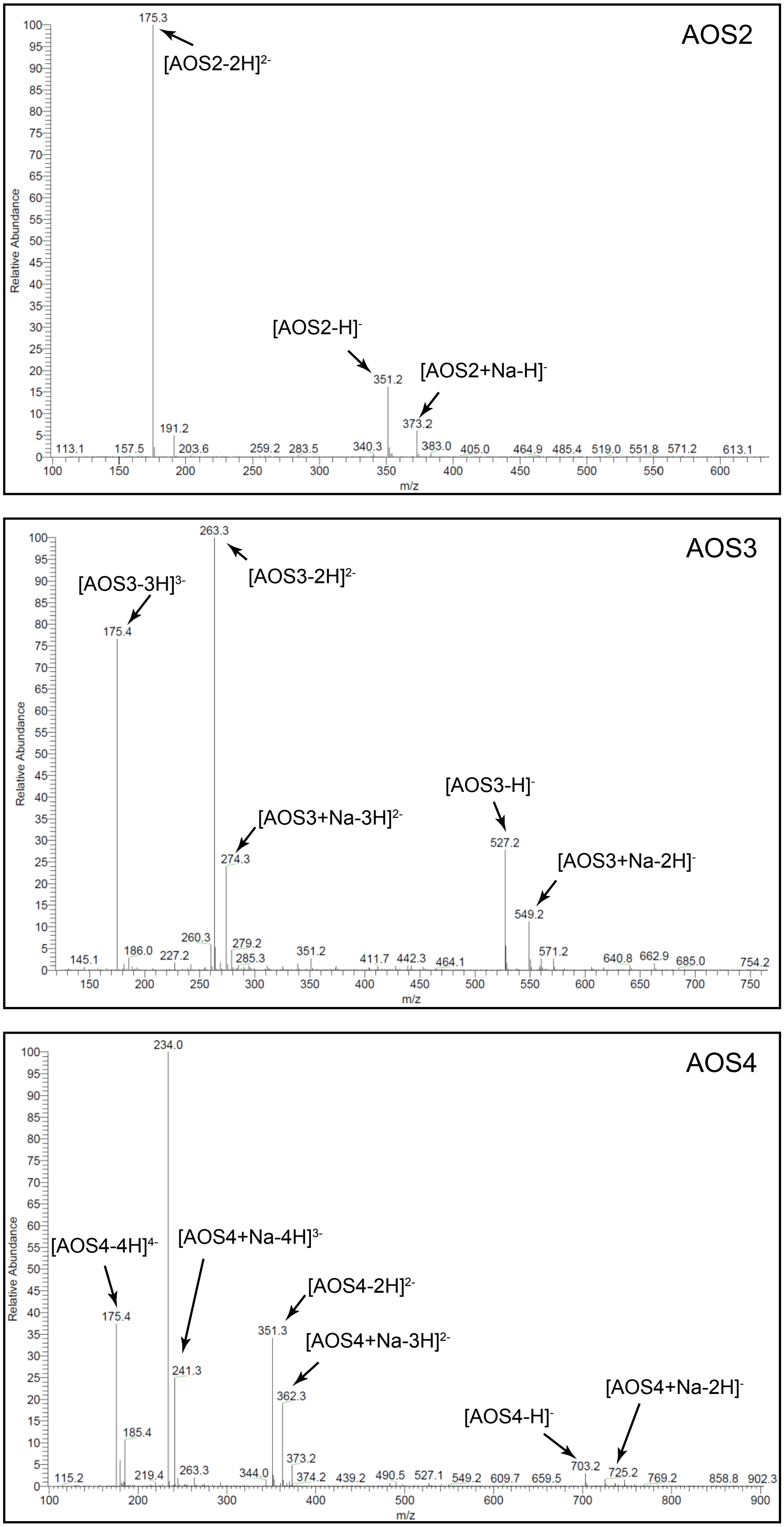

AOS2
[AOS2-2H]2-
[AOS2-H]-
[AOS2+Na-H]-
175.3
191.2
203.6
113.1
157.5
259.2
283.5
340.3
351.2
373.2
383.0
405.0
464.9
485.4
519.0
551.8
571.2
613.1
Relative Abundance
m/z
AOS3
[AOS3-3H]3-
[AOS3-2H]2-
[AOS3+Na-3H]2-
[AOS3-H]-
[AOS3+Na-2H]-
263.3
175.4
274.3
527.2
549.2
145.1
186.0
227.2
260.3
279.2
285.3
351.2
411.7
442.3
464.1
571.2
640.8
662.9
685.0
754.2
Relative Abundance
m/z
AOS4
[AOS4-4H]4-
[AOS4+Na-4H]3-
[AOS4-2H]2-
[AOS4+Na-3H]2-
[AOS4-H]-
[AOS4+Na-2H]-
234.0
175.4
241.3
351.3
362.3
185.4
373.2
703.2
725.2
115.2
219.4
263.3
344.0
374.2
439.2
490.5
527.1
549.2
609.7
659.5
769.2
858.8
902.3
Relative Abundance
m/z

Figure S2

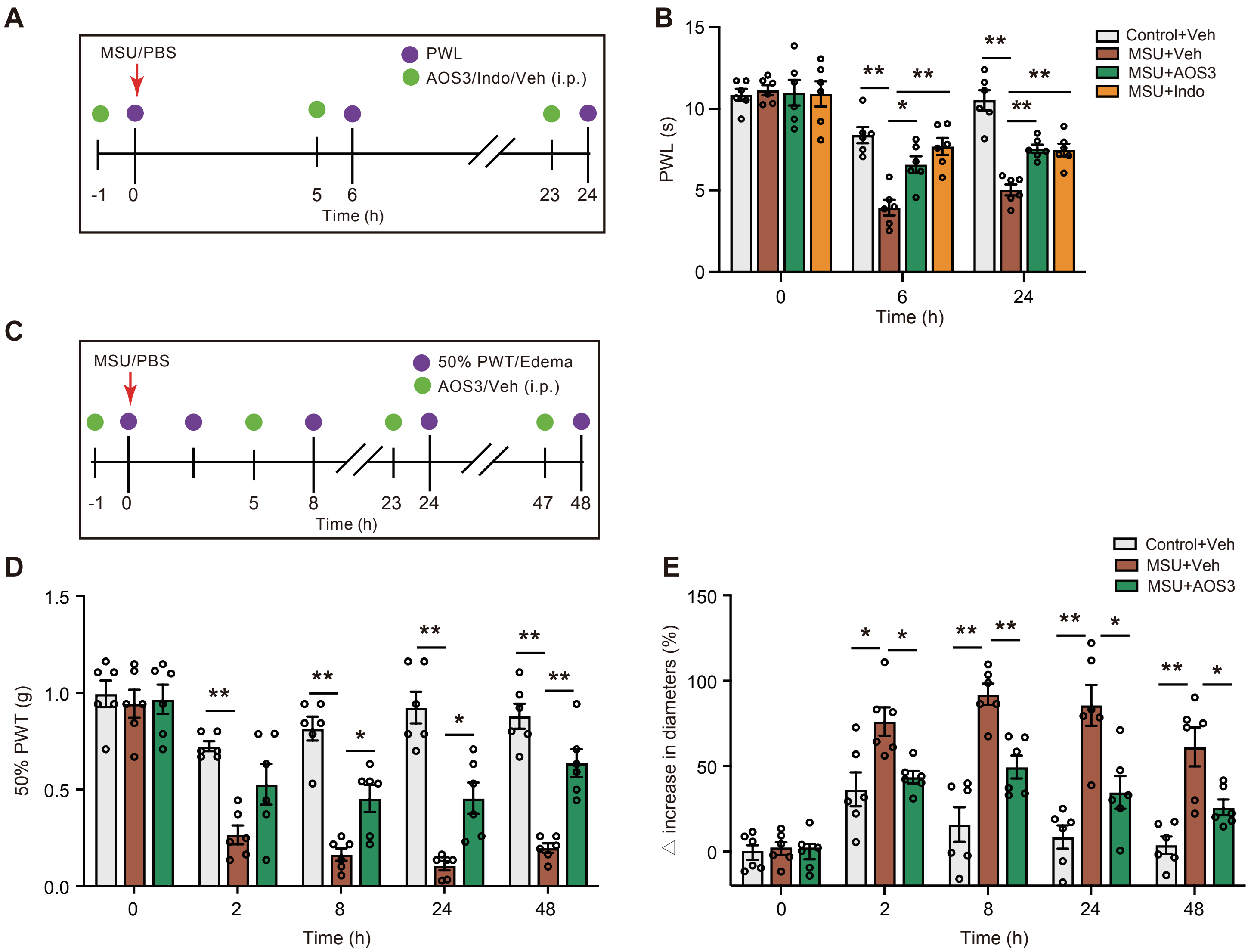
A
MSU/PBS
PWL
AOS3/Indo/Veh (i.p.)
-1 0 5 6 23 24
Time (h)
B
PWL (s)
Time (h)
Control+Veh
MSU+Veh
MSU+AOS3
MSU+Indo
C
MSU/PBS
50% PWT/Edema
AOS3/Veh (i.p.)
-1 0 5 8 23 24 47 48
Time (h)
D
50% PWT (g)
Time (h)
E
△ increase in diameters (%)
Time (h)
Control+Veh
MSU+Veh
MSU+AOS3

Figure S3

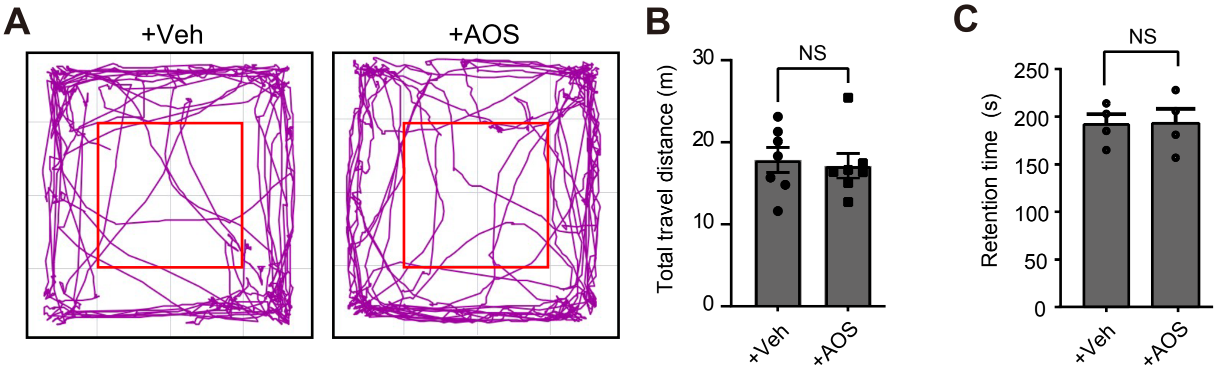
A
+Veh
+AOS
B
NS
30
20
10
0
Total travel distance (m)
+Veh
+AOS
C
NS
250
200
150
100
50
0
Retention time (s)
+Veh
+AOS

Figure S4

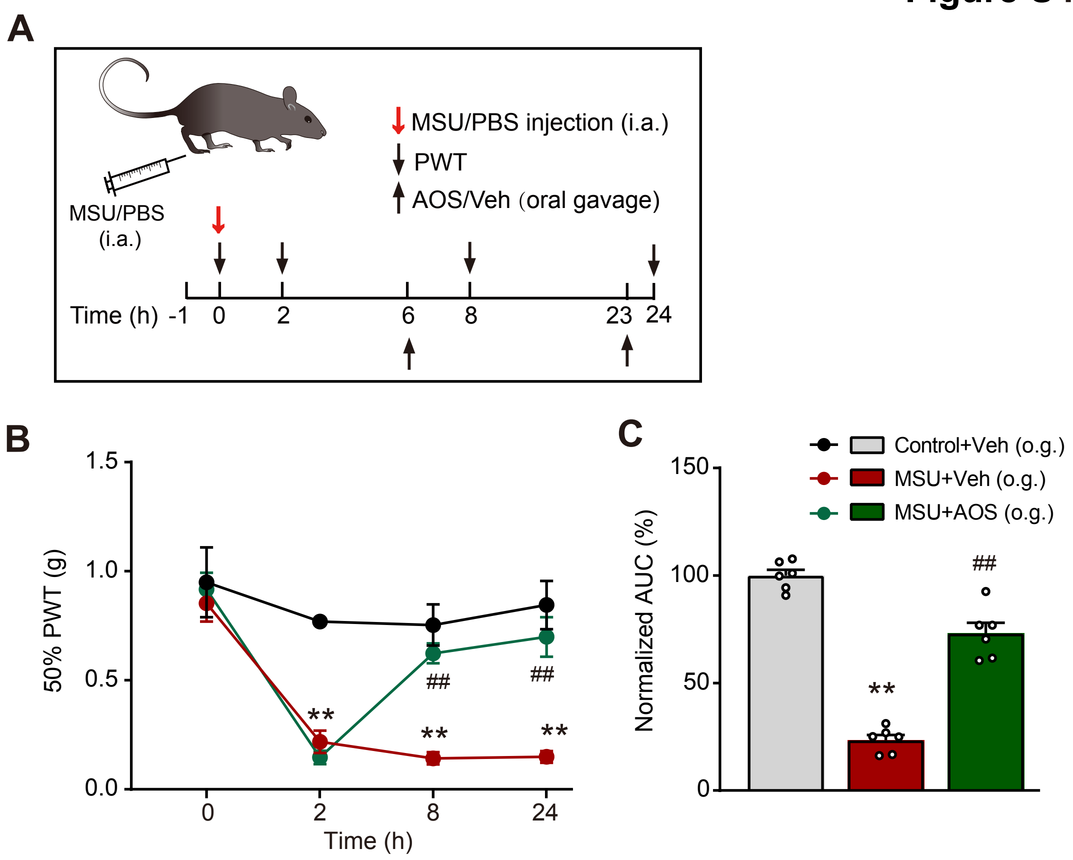

A
MSU/PBS injection (i.a.)
PWT
AOS/Veh (oral gavage)
MSU/PBS
(i.a.)
Time (h) -1 0 2 6 8 23 24
B
50% PWT (g)
1.5
1.0
0.5
0.0
0 2 8 24
Time (h)
**
##
C
Normalized AUC (%)
150
100
50
0
Control+Veh (o.g.)
MSU+Veh (o.g.)
MSU+AOS (o.g.)

Figure S5

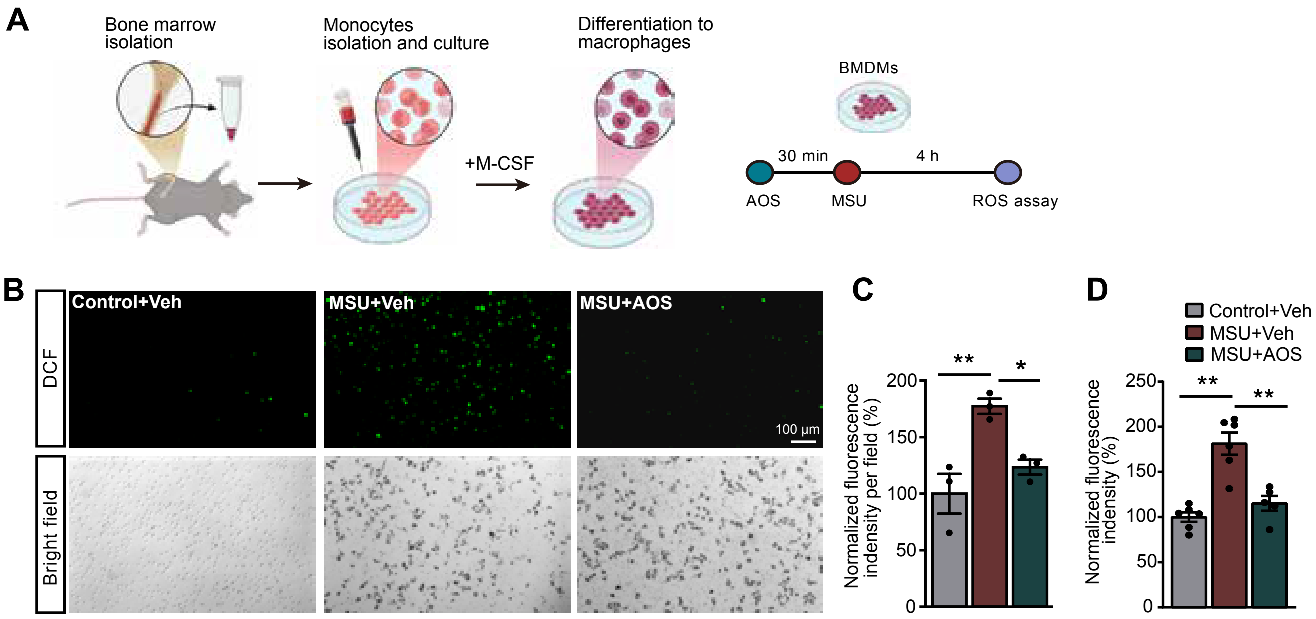

A
Bone marrow isolation
Monocytes isolation and culture
Differentiation to macrophages
+M-CSF
BMDMs
30 min
4 h
AOS
MSU
ROS assay
B
Control+Veh
MSU+Veh
MSU+AOS
DCF
Bright field
100 μm
C
Normalized fluorescence indensity per field (%)
200
150
100
50
0
**
*
D
Control+Veh
MSU+Veh
MSU+AOS
Normalized fluorescence indensity (%)
250
200
150
100
50
0
**
**

Figure S6

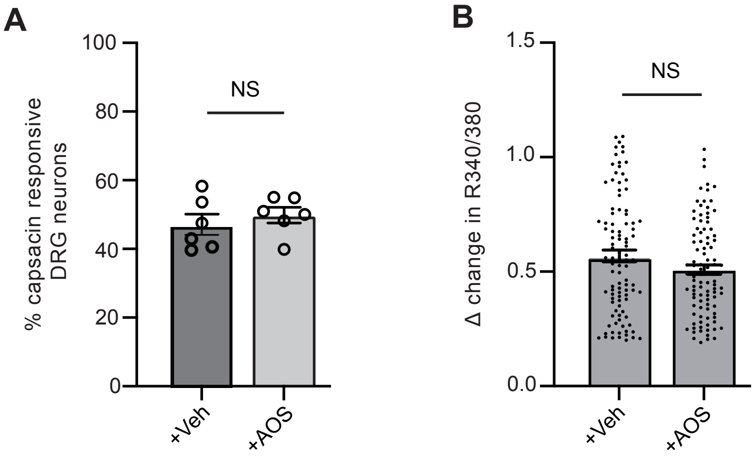
A
100
80
60
40
20
0
% capsacin responsive
DRG neurons
NS
+Veh
+AOS
B
1.5
1.0
0.5
0.0
Δ change in R340/380
NS
+Veh
+AOS
